# Long non-coding RNA RP11-197K6.1 as ceRNA promotes colorectal cancer progression via miR-135a-5p/DLX5 axis

**DOI:** 10.1186/s12967-024-05286-5

**Published:** 2024-05-17

**Authors:** Mingkun Wang, Xuben Niu, Maihuan Wang, Peng Zheng, Xiaoya Liu, Zhen Cao, Chaojun Zhang

**Affiliations:** 1 The Fifth School of Clinical Medicine, Navy Clinical College, Anhui Medical University, Hefei, Anhui 230032 China; 2 Department of General Surgery, School of Medicine, South China University of Technology, Guangzhou, 510006 China; 3 Department of General Surgery, The First Medical Center, Chinese PLA General Hospital, 28 Fuxing road, Haidian District, Beijing, 100853 China; 4 Department of General Surgery, The Sixth Medical Center of PLA General Hospital, 6 Fucheng road, Haidian District, Beijing, 100048 China

**Keywords:** Long noncoding RNA, miRNA, Colorectal cancer, RP11-197K6.1, miR-135a-5p, DLX5

## Abstract

**Background:**

Colorectal cancer (CRC) remains a major global health challenge, with high incidence and mortality rates. The role of long noncoding RNAs (lncRNAs) in cancer progression has received considerable attention. The present study aimed to investigate the function and mechanisms underlying the role of lncRNA RP11-197K6.1, microRNA-135a-5p (hsa-miR-135a-5p), and DLX5 in CRC development.

**Methods:**

We analyzed RNA sequencing data from The Cancer Genome Atlas Colorectal Cancer dataset to identify the association between lncRNA RP11-197K6.1 and CRC progression. The expression levels of lncRNA RP11-197K6.1 and DLX5 in CRC samples and cell lines were determined by real-time quantitative PCR and western blotting assays. Fluorescence in situ hybridization was used to confirm the cellular localization of lncRNA RP11-197K6.1. Cell migration capabilities were assessed by Transwell and wound healing assays, and flow cytometry was performed to analyze apoptosis. The interaction between lncRNA RP11-197K6.1 and miR-135a-5p and its effect on DLX5 expression were investigated by the dual-luciferase reporter assay. Additionally, a xenograft mouse model was used to study the in vivo effects of lncRNA RP11-197K6.1 on tumor growth, and an immunohistochemical assay was performed to assess DLX5 expression in tumor tissues.

**Results:**

lncRNA RP11-197K6.1 was significantly upregulated in CRC tissues and cell lines as compared to that in normal tissues, and its expression was inversely correlated with patient survival. It promoted the migration and metastasis of CRC cells by interacting with miR-135a-5p, alleviated suppression of DLX5 expression, and facilitated tumor growth.

**Conclusion:**

This study demonstrated the regulatory network and mechanism of action of the lncRNA RP11-197K6.1/miR-135a-5p/DLX5 axis in CRC development. These findings provided insights into the molecular pathology of CRC and suggested potential therapeutic targets for more effective treatment of patients with CRC.

**Supplementary Information:**

The online version contains supplementary material available at 10.1186/s12967-024-05286-5.

## Introduction

Colorectal cancer (CRC) poses a major threat to global health, particularly as a prominent malignancy of the digestive system [1]. With the aging population and lifestyle-related changes, the incidence and mortality rates of CRC continue to increase, which places a substantial burden on global public health systems. Despite considerable advancements in medical technology in recent years, including early screening, improvements in surgical techniques, and the application of targeted therapies and immunotherapies, the treatment outcomes for patients with advanced or metastatic CRC remain poor [2]. This is primarily attributed to tumor recurrence and metastasis of CRC and the lack of effective predictive biomarkers and therapeutic targets. Therefore, a deeper understanding of the molecular pathogenesis of CRC and the identification of novel biomarkers and therapeutic targets are crucial to improve the survival rates of CRC patients.

In the field of cancer biology, long noncoding RNAs (lncRNAs) have emerged as a hot topic of research. lncRNAs are a class of RNA molecules longer than 200 nucleotides that do not encode proteins [3], nevertheless, they play key roles in gene expression regulation, participate in cellular biological processes, and influence tumor development and metastasis [4]. Recent studies have shown that lncRNAs were closely associated with the occurrence, progression, and metastasis of various cancers [5]. For instance, the overexpression of lncRNA HUMT is related to lymph node metastasis in breast cancer [6], the downregulation of lncRNA ZNRD1-AS1 promotes tumor proliferation, migration, and angiogenesis in lung cancer [7], and the expression of lncRNA HOTAIR indicates metastasis in CRC [8]. However, the biological mechanisms and clinical significance of lncRNAs in CRC regulation remain largely unknown.

Some studies suggest that lncRNAs may act as competing endogenous RNAs (ceRNAs) and regulate the biological functions or expression of microRNAs (miRNAs) [9–12]. For example, lncRNA CTSLP8 enhances ovarian cancer metastasis through the miR-199a-5p/CTSL1 signaling axis [13]. Additionally, lnc-GAN1 inhibits the progression of non-small cell lung cancer (NSCLC) by sponging miR-26a-5p to activate PTEN [14]. The clinical characteristics and cellular mechanisms of miRNAs in CRC regulation require further investigation, as their biological mechanisms and clinical significance in CRC remain largely unexplored.

The DLX5 (Distal-Less Homeobox 5) gene plays an indispensable role in embryogenesis and development [15]. Previous studies have shown that DLX5 was upregulated in various tumors [16]. DLX5 can promote ovarian cancer cell proliferation by enhancing the IRS-2-AKT pathway; moreover, in osteosarcoma, DLX5 can activate the NOTCH signaling pathway to promote tumor progression [17]. According to previous research, the overexpression of DLX5 can promote tumor cell growth by directly targeting the c-MYC gene [18]. However, the role and expression of DLX5 in CRC have not yet been reported.

Herein, we identified lncRNA RP11-197K6.1, which is associated with CRC development. By screening for differentially expressed lncRNAs between CRC tissues and normal tissues, we identified potential predictive biomarkers for CRC based on the lncRNA/miRNA axis. Although various lncRNAs may act as potential tumor suppressors or oncogenes in CRC, we focused on the function of lncRNA RP11-197K6.1. This lncRNA showed high expression in CRC tissues and cell lines, and its expression was associated with poor prognosis. In vivo and in vitro experiments demonstrated that lncRNA RP11-197K6.1 promoted the progression of CRC. We also elucidated the ceRNA regulatory mechanism of the lncRNA RP11-197K6.1/miR-135a-5p axis in the DLX5-mediated progression and growth of CRC in vivo. Our study provided new insights into the mechanisms of CRC metastasis and promising therapeutic targets for treating CRC.

## Materials and methods

### Human tissue samples

Tissue samples, including 20 colorectal cancer (CRC) cases and 20 normal colon tissues, were collected from the Sixth Medical Center of the Chinese PLA General Hospital. Inclusion criteria were as follows: (1) histologically confirmed CRC; (2) no prior anti-tumor treatment with complete clinicopathological data and follow-up information. Exclusion criteria included a history of cardiovascular disease, infectious diseases, alcoholism, mental illness, or other malignancies. Tissue specimens were immediately frozen at -80 °C. CRC was graded and staged according to the World Health Organization (WHO) criteria and the American Joint Committee on Cancer (AJCC) TNM staging system, 7th edition, respectively. The study was approved by the Ethics Committee of the Sixth Medical Center of the Chinese PLA General Hospital (Approval No. HZKY-PJ-2023-57) and conducted in accordance with the ethical guidelines of the Declaration of Helsinki. Written informed consent was obtained from all participants.

### Cell culture

The HCT116, HT-29, LOVO, SW480, and NCM460 cell lines were obtained from the American Type Culture Collection (ATCC), USA. To ensure proper growth and maintenance of phenotypic characteristics, each cell line was cultured under its specific optimal conditions as recommended by ATCC. HCT116 and HT-29 cells were cultured in McCoy’s 5 A Medium modified (ATCC, USA), supplemented with 10% fetal bovine serum (FBS), at 37 °C in a humidified atmosphere containing 5% CO2. LOVO cells were grown in F-12 K Medium (ATCC, USA) with 10% FBS, and NCM460 cells were cultured in M3:10™ Medium (INCELL Corporation, USA) specifically designed to support their normal phenotype, at 37 °C in an atmosphere of 5% CO2. SW480 cells were maintained in Leibovitz’s L-15 Medium (ATCC, USA) supplemented with 10% FBS, at 37 °C in a non-CO2 environment (100% Air). These specific culture conditions are critical to ensure that the cells express relevant phenotypes and that gene expression and transcription processes are not adversely affected.

### Plasmid construction and cell transfection

HCT116 and SW480 cells (2 × 10^5) were seeded in 6-well plates and cultured overnight. Transfection reagents included si-LncRNA-RP11-197K6.1 (small interfering RNA targeting LncRNA RP11-197K6.1), si-NC (small interfering RNA negative control), miR-135a-5p mimic (synthetic mimic of microRNA-135a-5p), NC mimic (negative control mimic), miR-135a-5p inhibitor (synthetic inhibitor of microRNA-135a-5p), NC inhibitor (negative control inhibitor), and pcDNA3.1-DLX5 (plasmid pcDNA3.1 containing DLX5 gene), all purchased from Shanghai Jima Industrial Co., Ltd. Transfections were performed using Lipofectamine 2000 reagent (Invitrogen, Carlsbad, CA, USA) following the manufacturer’s instructions. Sequences for miR-135a-5p and miR-135a-5p inhibitor are listed in Supplementary Table S1.

### RNA isolation and real-time quantitative PCR

RNA was extracted from cells using TRIzol reagent (Invitrogen/Thermo Fisher Scientific). To ensure the integrity and quality of RNA, samples were assessed using a NanoDrop spectrophotometer (Thermo Fisher Scientific) for concentration and purity, and RNA integrity was confirmed by agarose gel electrophoresis. Complementary DNA (cDNA) was synthesized from lncRNA and mRNA using the PrimeScript™ RT Reagent Kit (TaKaRa Bio, Kusatsu, Japan). miRNA reverse transcription was performed using the miRNA Reverse Transcription Kit (TaKaRa Bio). For real-time quantitative PCR (RT-qPCR), the TB Green Fast qPCR Mix (TaKaRa Bio) was utilized, with GAPDH and U6 serving as internal controls for mRNA and miRNA, respectively. The thermal cycling conditions were as follows: initial denaturation at 95 °C for 30 s, followed by 40 cycles of 95 °C for 5 s and 60 °C for 10 s, with a final hold at 4 °C. The 2-ΔΔCt method was used for data analysis, allowing for relative quantification of gene expression. Primer sequences for all targets and controls are listed in Supplementary Table S2.

### Western blot

Total protein from CRC cells and tissues was extracted using RIPA lysis buffer (MedChemExpress, Monmouth Junction, NJ, USA) containing phosphatase and protease inhibitors. Protein concentration was determined using the BCA Protein Assay Kit (Beyotime, Shanghai, China). Proteins were separated by 10% SDS-PAGE and transferred to polyvinylidene difluoride (PVDF) membranes (Millipore, Burlington, MA, USA). Membranes were blocked with 5% non-fat milk at room temperature for 2 h and incubated with primary antibodies overnight at 4 °C. Primary antibodies used were anti-DLX5 (1:1,000, Affinity Biosciences, Cat#DF3220), anti-GAPDH (1:10,000, Affinity Biosciences, Cat#AF7021), anti-ACTB (1:10,000, Affinity Biosciences, Cat#AF7018), anti-caspase-3 (1:1,000, MedChemExpress, Cat#HY-P80046), anti-cleaved-caspase-3 (1:1,000, MedChemExpress, Cat#HY-P80623), anti-E-cadherin (1:1,000, Affinity Biosciences, Cat#AF0131), anti-Vimentin (1:1,000, Affinity Biosciences, Cat#AF7013), anti-Cyclin D1 (1:1,000, Affinity Biosciences, Cat#AF0931), anti-Cyclin E (1:1,000, Affinity Biosciences, Cat#AF0144), anti-Cyclin A2 (1:1,000, Abcam, ab181591), anti-Integrin alpha V (1:1,000, Abcam, ab179475), anti-caspase-7 (1:1,000, Affinity Biosciences, Cat#DF6441), anti-cleaved-caspase-8 (Asp384) (1:1,000, Affinity Biosciences, Cat#AF5267), anti-cleaved-caspase-9 (Asp353) (1:1,000, Affinity Biosciences, Cat#AF5240), anti-PCNA (1:10,000, ProteinTech, Cat#10205-2-AP), and anti-MMP9 (1:1,000, Affinity Biosciences, Cat#AF5228). After incubation with corresponding secondary antibodies for 1.5 h at room temperature, protein bands were detected using an ECL detection kit (Beyotime, Shanghai, China).

### Subcellular fractionation and fluorescence in situ hybridization

LncRNA-RP11-197K6.1 in the nucleus and cytoplasm was separated using the NE-PER Nuclear and Cytoplasmic Extraction Reagent (Invitrogen, Carlsbad, CA, USA) and quantified by RT-qPCR. For fluorescence in situ hybridization (FISH) experiments, cells were fixed, permeabilized, and hybridized with a 20 µM Cy5-labeled LncRNA-RP11-197K6.1 probe mixture. Nuclei were stained with DAPI (Sigma-Aldrich, St. Louis, MO, USA), and the distribution of LncRNA-RP11-197K6.1 within cells was observed using a fluorescence microscope (NIKON Eclipse Ti, Japan).

### Luciferase reporter assay

The 3’UTR of LncRNA-RP11-197K6.1 and its mutant containing the miR-135a-5p binding site were cloned into the pGL3 luciferase reporter vector (Invitrogen, Carlsbad, CA, USA). HCT116 cells (5 × 10^3) were seeded in 96-well plates and co-transfected with miR-135a-5p mimic and either wild-type p-GL3-LncRNA-RP11-197K6.1 (LncRNA-RP11-197K6.1-WT) or mutant p-GL3-LncRNA-RP11-197K6.1 (LncRNA-RP11-197K6.1-Mut) vectors. Luciferase activity was measured 48 h post-transfection using the Dual-Luciferase Reporter Assay System (Promega, Madison, WI, USA) and normalized to Renilla luciferase activity driven by a constitutive promoter (phRL vector).

### Cell proliferation assay

Approximately 2 × 10^3 cells were seeded in 96-well plates and cell proliferation was assessed at 0, 24, 48, 72, and 96 h using the Cell Counting Kit-8 (CCK-8, Solarbio, Beijing, China). Cell growth curves were plotted based on the absorbance values at each time point.

### Wound healing assay

HCT116 and SW480 cells were seeded in 6-well plates and grown to sub-confluent density. After serum starvation in DMEM (Gibco, Thermo Fisher Scientific, Inc.) for 24 h, a straight line wound was created at the bottom of the plate using a 10 µl sterile pipette tip. After gentle washing, cells were cultured in serum-free medium for 24 h. Cell migration was observed and calculated at 0 and 24 h using an inverted microscope. Wound healing (%) = (initial wound area - final wound area) ÷ initial wound area × 100.

### Migration and invasion assays

Migration and invasion assays were performed using 24-well Transwell plates (CORNING-COSTAR). A total of 5 × 10^4 cells suspended in serum-free medium were seeded into the upper chamber, which was either uncoated or coated with Matrigel. Complete medium containing 10% FBS was added to the lower chamber. After 24 h of incubation at 37 °C, non-invading cells in the upper chamber were removed, and invading cells were fixed with 4% formaldehyde and stained with crystal violet. Cells were observed and counted under a microscope (NIKON ECLIPSE C1, Japan).

### Annexin V/7-AAD flow cytometry assay

After various treatments, collect colorectal cancer cells and wash them once with pre-chilled PBS at 4°C. Suspend cells in 100 μl of binding buffer (BD Biosciences, USA, Cat No: 556454). Add Annexin V-PE (BD Biosciences, USA, Cat No: 556422) and 7-AAD (BD Biosciences, USA, Cat No: 559925) to each sample. Incubate samples in the dark at room temperature for 30 minutes to ensure proper dye binding. Analyze apoptotic cell population using a flow cytometer (FACS CANTO II, Becton Dickinson, France). Calibrate the instrument according to the manufacturer’s recommendations and analyze data using FlowJo software (Version 10.0.7r2, FlowJo LLC, Tree Star, Ashland, OR, USA). Cells stained with Annexin V-PE are defined as early apoptotic cells, and cells positive for both Annexin V-PE and 7-AAD are defined as late apoptotic cells.

### Xenograft mouse model

Female BALB/c nude mice (6–8 weeks old, SPF (Beijing) Biotechnology, Beijing, China) were used for experiments and randomly divided into 4 groups (*n* = 6 per group). HCT116 cells (1 × 10^6) were subcutaneously injected into the back of each mouse. Mice were euthanized 14 days post-injection, and tumors were excised, weighed, and the volume was calculated using the modified ellipsoid formula (V = [length ÷ 2] × width^2). Tumors were fixed in 4% formaldehyde and embedded in paraffin for immunohistochemical staining. Animal experiments were conducted in accordance with institutional guidelines and approved by Institutr Of Analysis And Testing, Beijing Academy Of Science And Technology(Beijing Center for Physical&Chemical Analysis)Application for Laboratory Animal Welfare and Ethical review (Approval No. 231,220-SWDWF-004).

### Immunohistochemistry

Tissue sections were treated with 3% hydrogen peroxide and incubated in 5% goat serum. Consecutive tissue sections were incubated overnight with anti-DLX5 (1:200, Affinity Biosciences Cat# DF3220) or anti-KI67 (1:200, Affinity Biosciences Cat# AF0198) antibodies, followed by incubation with biotinylated secondary antibodies. Before microscopic analysis, sections were treated with streptavidin-peroxidase complex and stained with 3,3’-diaminobenzidine (Maishin Bio, Fuzhou, China).

### TUNEL assay

TUNEL assay was performed by using Colorimetric TUNEL Apoptosis Assay Kit (Beyotime, China), Initially, paraffin-embedded tissue sections were deparaffinized and rehydrated through a graded series of alcohols. The sections were then treated with proteinase K to expose DNA ends. Following this, TUNEL staining was performed according to the manufacturer’s instructions to label DNA breaks. Finally, the sections were observed and imaged under a light microscope (Nikon, Japan) to assess the extent of apoptosis.

### Bioinformatics analysis

LncRNA expression changes were explored through the TCGA-COAD database, and data were downloaded and processed using the R package TCGAbioLinks. Differentially expressed lncRNAs were identified, and the survival impact of RP11-197K6.1 in colon cancer was analyzed using the Kaplan-Meier method. Pearson correlation analysis was used to evaluate the correlation between RP11-197K6.1 and DLX5. Gene set enrichment analysis (GSEA) was conducted to investigate the potential biological functions of DLX5. All statistical analyses were performed using R, and graphs were generated using the ggplot2 package. Additionally, miRNA targets of RP11-197K6.1 and DLX5 were predicted using miRCode and ENCORI, and potential binding sites with miR-135a-5p were predicted using TargetScan. These predictions will be further validated in subsequent experiments.

### Statistical analysis

Data are presented as mean ± standard deviation (SD). Statistical analysis was performed using SPSS 20.0 software (IBM Corporation, Armonk, NY, USA), including the t-test for comparisons between two independent groups and one-way analysis of variance (ANOVA) for data involving more than two independent groups. The significance level for all statistical tests was set at *P* < 0.05, considered to indicate statistically significant differences. Graphical presentations were created using GraphPad Prism 9.0 software (GraphPad Software, San Diego, CA, USA) to clearly display the statistical results.

## Results

### High expression of LncRNA-RP11-197K6.1 in CRC tissues and cell lines

**Fig. 1:**
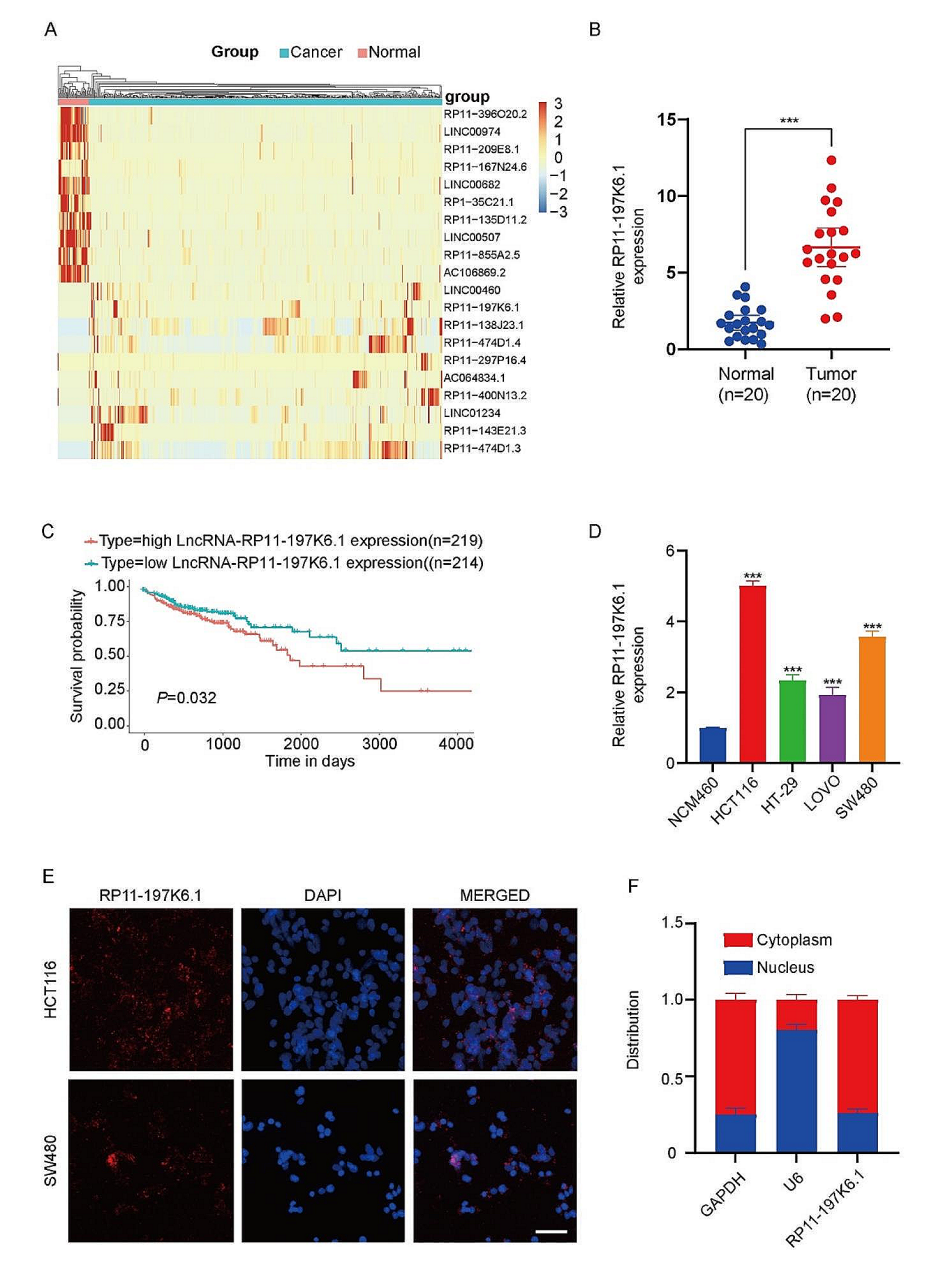
Comprehensive analysis of lncRNA RP11-197K6.1 expression in CRC tissues and cell lines. **A**: Comparison of lncRNA RP11-197K6.1 expression between CRC tissues and normal tissues based on the TCGA-COAD dataset. **B**: RT-qPCR quantification of lncRNA RP11-197K6.1 expression in 20 pairs of CRC tissues and adjacent normal tissues. **C**: Kaplan-Meier plots showing correlation of high vs. low lncRNA RP11-197K6.1 expression level with survival rates in CRC patients. **D**: lncRNA RP11-197K6.1 expression levels across different CRC cell lines (HCT116, HT-29, LOVO, and SW480) as compared to that in the control cell line NCM460. **E**: Subcellular localization of lncRNA RP11-197K6.1 in HCT116 and SW480 cells demonstrated by FISH analysis (scale = 50 μm). **F**: Results of RT-qPCR analysis for lncRNA RP11-197K6.1 nuclear-cytoplasmic distribution in HCT116 cells, showing the distribution of lncRNA RP11-197K6.1 between the nucleus and cytoplasm (***P < 0.001, *n* = 3).

To determine the role of lncRNA RP11-197K6.1 in CRC, we analyzed the RNA sequencing (RNA-seq) profiles of CRC tissues and normal tissues from The Cancer Genome Atlas Colorectal Cancer (TCGA-COAD) dataset. As shown in Fig. 1A, lncRNA RP11-197K6.1 exhibited higher expression levels in CRC tissues than in normal tissues. Validation by RT-qPCR revealed that lncRNA RP11-197K6.1 expression was significantly upregulated in CRC cell lines (Figure S1A). We also collected matched fresh CRC tissues and normal adjacent tissues from patients who underwent surgery. To investigate the relationship between lncRNA RP11-197K6.1 expression and clinicopathological characteristics, patients were categorized into two groups according to their lncRNA RP11-197K6.1 expression levels. The clinical features of these groups were then analyzed using the chi-square test. As illustrated in Table 1, the lncRNA RP11-197K6.1 expression levels were significantly correlated with the depth of tumor invasion (*P* = 0.009), lymph node metastasis (*P* = 0.013), and AJCC stage (*P* = 0.011). The expression levels of lncRNA RP11-197K6.1 in 20 pairs of CRC tissues and adjacent normal tissues were quantified by RT-qPCR. The results showed that lncRNA RP11-197K6.1 was upregulated in CRC tissues (Fig. 1B). The Kaplan-Meier analysis of the TCGA-COAD dataset showed that patients with a higher expression level of lncRNA RP11-197K6.1 had significantly worse survival rates (Fig. 1C). We also analyzed the expression of lncRNA RP11-197K6.1 in CRC cell lines. Compared to the control cell line NCM460, the CRC cell lines HCT116, HT-29, LOVO, and SW480 showed a significant increase in the lncRNA RP11-197K6.1 expression level (Fig. 1D).

**Table 1:** Correlation between lncRNA RP11-197K6.1 expression and clinicopathological characteristics of CRCs in human CRC tissues

Characteristics	Case(*n* = 20)	Expression of RP11-197K6.1		*P* value
		Low(*n* = 10)	High(*n* = 10)	
Age				
≤ 60	11	6	5	1.000
>60	9	4	5	
Sex				
Female	14	6	8	0.626
Male	6	4	2	
Tumor location				
Right colon	6	4	2	0.147
Left colon	8	5	3	
Rectum	6	1	5	
AJCC stage				
Stage I	2	2	0	0.011
Stage II	5	5	0	
Stage III	6	2	4	
Stage IV	7	1	6	
LN metastasis				
Yes	13	4	9	0.013
No	7	7	0	
Tumor invasion				
T1	2	2	0	0.009
T2	1	1	0	
T3	10	7	3	
T4	7	0	7	

Because the function of lncRNAs is associated with their subcellular localization, we performed nuclear-cytoplasmic separation experiments in CRC cell lines. RT-qPCR analysis of lncRNA RP11-197K6.1 expression in the nucleus and cytoplasm showed that lncRNA RP11-197K6.1 was primarily expressed in the cytoplasm of HCT116 and SW480 cells; this finding was confirmed by FISH analysis (Fig. 1E, F). Thus, lncRNA RP11-197K6.1 was highly expressed in CRC tissues and cell lines, was predominantly localized in the cytoplasm of CRC cells, and was associated with patient survival rates.

### Knockdown of lncRNA RP11-197K6.1 promotes apoptosis and inhibits the proliferation, migration, and invasion of CRC cells

**Fig. 2:**
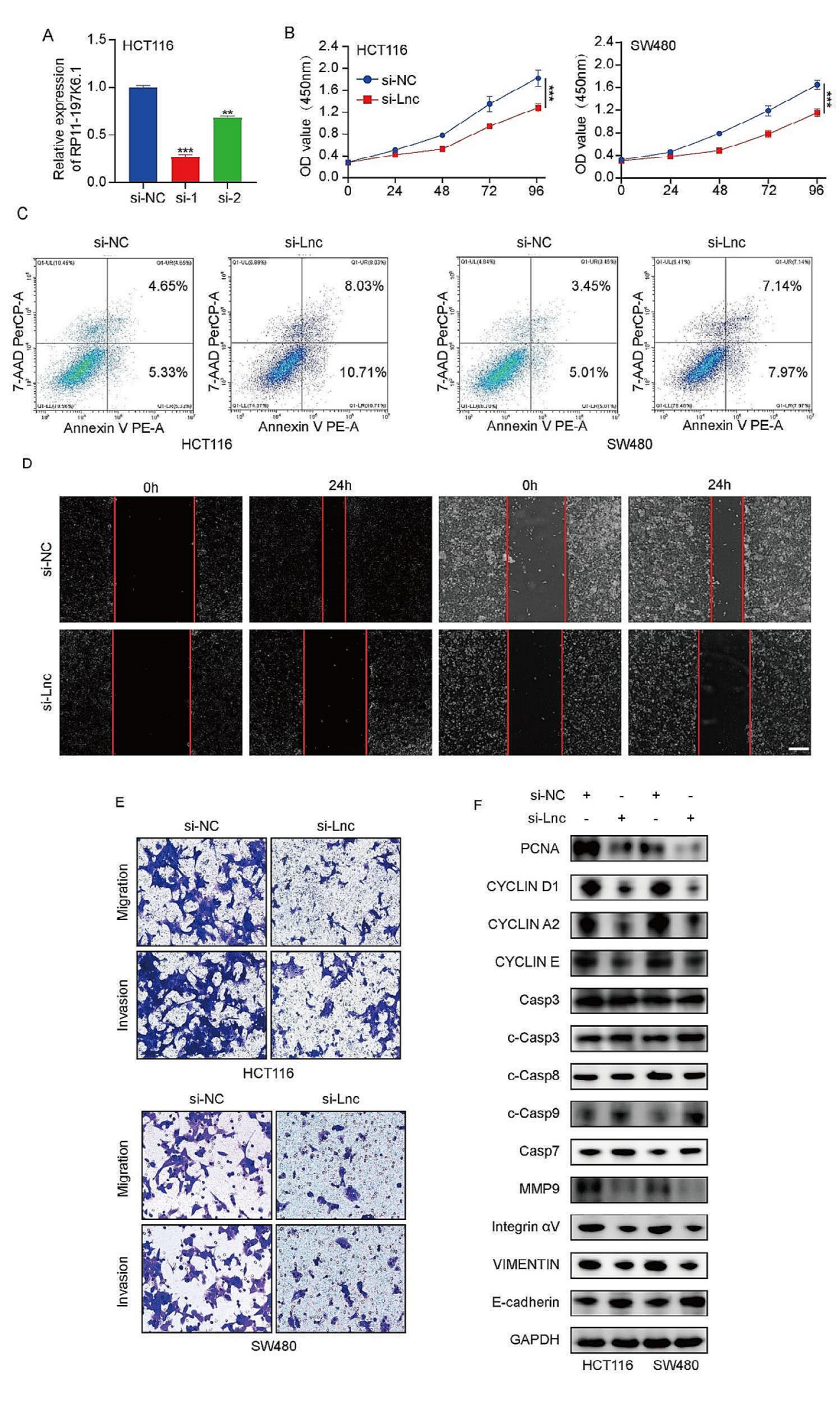
Functional effects of lncRNA RP11-197K6.1 knockdown in CRC cells. **A**: Confirmation of lncRNA RP11-197K6.1 knockdown efficiency in HCT116 cells by RT-qPCR. **B**: Reduction in the proliferation of HCT116 and SW480 cells post-knockdown, as determined by proliferation assays. **C**: Increase in the apoptosis of HCT116 and SW480 cells following lncRNA RP11-197K6.1 knockdown, as measured by flow cytometry. **D** & **E**: Decrease in the migration and invasion of HCT116 and SW480 cells post-knockdown, as assessed by wound healing (scale = 200 μm) and Transwell assays (scale = 50 μm), respectively. 2 *F*: Changes in the levels of proteins related to the proliferation, apoptosis, and migration of HCT116 and SW480 cells after lncRNA RP11-197K6.1 knockdown, as analyzed by western blotting assay (**P < 0.01, ***P < 0.001, *n* = 3)

To investigate the function of lncRNA RP11-197K6.1 in CRC, we screened multiple siRNAs to reduce the expression of lncRNA RP11-197K6.1 in the cell lines. We used HCT116 and SW480 cell lines to confirm the knockdown efficiency of the siRNAs targeting lncRNA RP11-197K6.1 (Fig. 2A, Supplementary S1B). Functionally, the knockdown of lncRNA RP11-197K6.1 inhibited the proliferation of HCT116 and SW480 cells (Fig. 2B) and promoted their apoptosis (Fig. 2C). The downregulation of lncRNA RP11-197K6.1 expression significantly inhibited the migration and invasion of HCT116 and SW480 cells, as demonstrated by the results of wound healing and Transwell assays (Fig. 2D, E). We analyzed the levels of proliferation-, apoptosis-, and migration-related proteins after lncRNA RP11-197K6.1 knockdown. The knockdown significantly affected the protein levels of proliferating cell nuclear antigen (PCNA), with a similar effect on the proliferation markers such as Cyclin D1, Cyclin A2, and Cyclin E. Among the migration-related proteins, matrix metalloproteinase 9 (MMP9) expression levels were altered, consistent with changes observed in the expression levels of vimentin and integrin αV, whereas E-cadherin expression showed the opposite trend. Additionally, lncRNA RP11-197K6.1 knockdown increased the levels of apoptosis-related proteins, including cleaved caspase 3, caspase 7, and cleaved caspase 9, thus suggesting the activation of intrinsic apoptotic pathways. Notably, no significant change was observed in the expression level of cleaved caspase 8 (Fig. 2F). Thus, lncRNA RP11-197K6.1 regulated the development of CRC, inhibited the apoptosis of CRC cells, and promoted the proliferation, migration, and invasion of CRC cells.

### DLX5 is upregulated in CRC cells and positively correlated with lncRNA RP11-197K6.1 expression

**Fig. 3:**
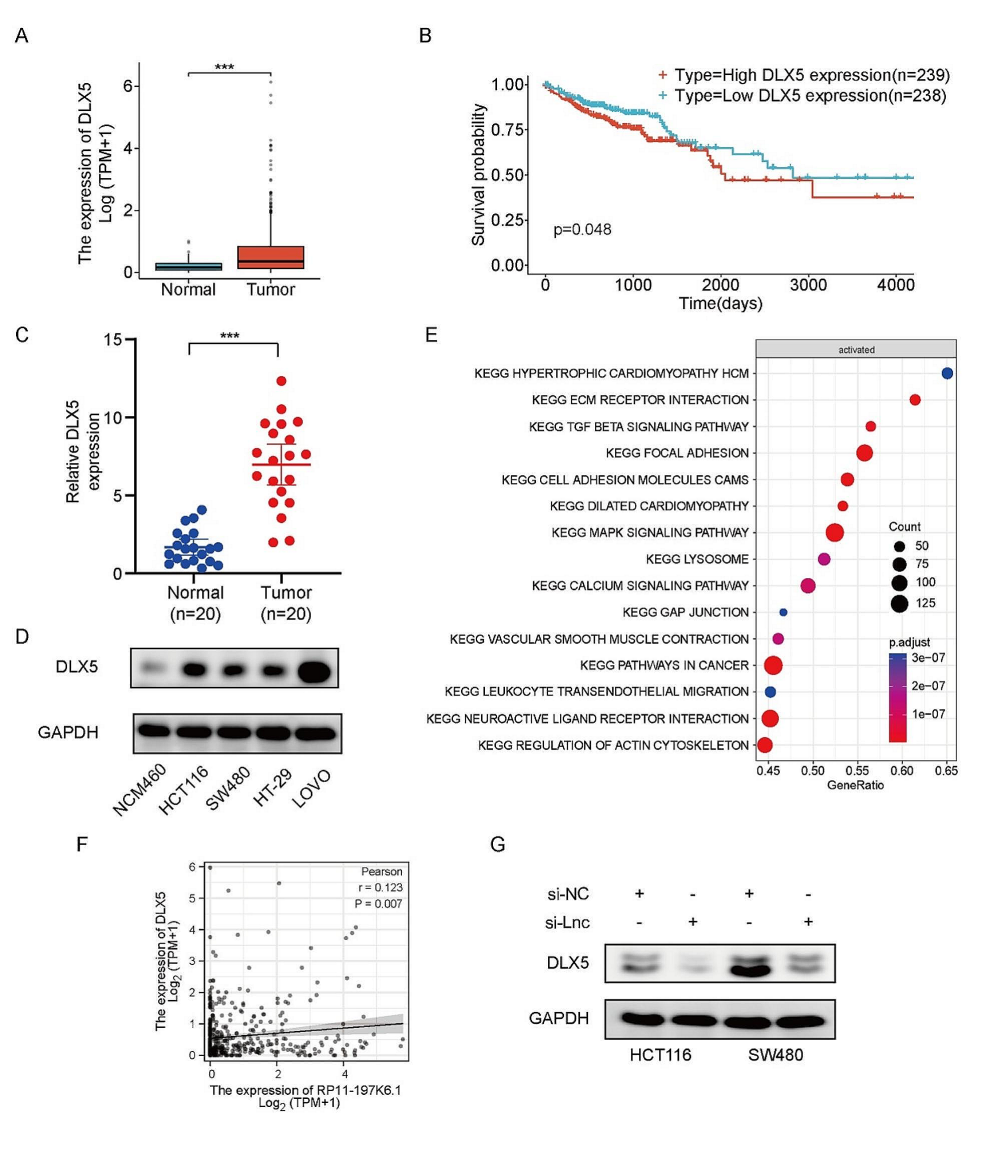
DLX5 expression analysis and its association with CRC progression. **A**: Comparison of DLX5 expression levels between CRC tissues and normal tissues based on the TCGA-COAD dataset. **B**: Kaplan-Meier survival curves for comparison of survival rates between CRC patients with high vs. low DLX5 expression. **C**: RT-qPCR analysis of DLX5 expression in matched fresh CRC tissues and adjacent normal tissues collected from patients during surgery. **D**: Comparison of DLX5 expression levels across various CRC cell lines (HCT116, HT-29, LOVO, and SW480) and the control cell line NCM460. **E**: Gene set enrichment analysis indicated significant enrichment of hypertrophic cardiomyopathy-related genes, the extracellular matrix-receptor interaction pathway, and the TGF-β signaling pathway in CRC patients with high DLX5 expression, thus suggesting the association of these pathways with DLX5 upregulation. **F**: Pearson’s correlation analysis showed a positive correlation between the expression levels of lncRNA RP11-197K6.1 and DLX5 in CRC tissues. **G**: Western blotting assays showed a decrease in DLX5 expression following lncRNA RP11-197K6.1 knockdown in HCT116 and SW480 cells (***P < 0.001, *n* = 3).

To further investigate the role of DLX5 in CRC cells and its interaction with lncRNA RP11-197K6.1, this study used RNA sequencing data from the TCGA dataset for a preliminary analysis. DLX5 was significantly overexpressed in CRC tissues as compared to that in normal tissues (Fig. 3A). This result suggested that DLX5 may play a key role in CRC development. The Kaplan-Meier survival curve analysis further revealed that CRC patients with a high DLX5 expression level had a poor survival rate (Fig. 3B), thus confirming that DLX5 could be a potential adverse prognostic marker.

To validate the results of the preliminary analysis of the TCGA dataset, we detected the expression levels of DLX5 in 20 pairs of freshly resected CRC tissues and their adjacent normal tissues by RT-qPCR. The results showed that the DLX5 expression level was significantly increased in CRC tissues as compared to that in adjacent normal tissues (Fig. 3C); this finding was consistent with the results of the dataset analysis. Additionally, we evaluated the expression of DLX5 in multiple CRC cell lines. DLX5 expression was significantly upregulated in CRC cell lines, including HCT116, HT-29, LOVO, and SW480 (Fig. 3D), as compared to that in the normal colon cell line NCM460.

To further determine the biological function of DLX5, we divided CRC patient samples from the TCGA dataset into high and low expression groups according to the DLX5 expression levels and used gene set enrichment analysis (GSEA) to assess the characteristic signaling pathways in patients with high DLX5 expression. The results revealed that the pathways related to tumor progression, such as hypertrophic cardiomyopathy-related genes, the extracellular matrix-receptor interaction pathway, and the TGF-β signaling pathway, were significantly enriched in the high DLX5 expression group (Fig. 3E). This finding implied that DLX5 may promote the development of CRC by participating in specific signaling pathways.

By performing Pearson’s correlation analysis, we found that the expression levels of lncRNA RP11-197K6.1 and DLX5 were positively correlated in CRC tissues (Fig. 3F). Additionally, after the knockdown of lncRNA RP11-197K6.1 in HCT116 and SW480 cells, western blotting assay showed a corresponding downregulation in the protein levels of DLX5 (Fig. 3G); this finding further confirmed the positive regulatory relationship between lncRNA RP11-197K6.1 and DLX5.

These results indicated the high expression of DLX5 in CRC and its positive correlation with lncRNA RP11-197K6.1, thus suggesting that DLX5 may promote CRC development by participating in specific biological signaling pathways and forming a mutual regulatory network with lncRNA RP11-197K6.1, thereby collectively influencing the progression and prognosis of CRC.

### lncRNA RP11-197K6.1 binds to miR-135a-5p and acts as a ceRNA in CRC cells

**Fig. 4:**
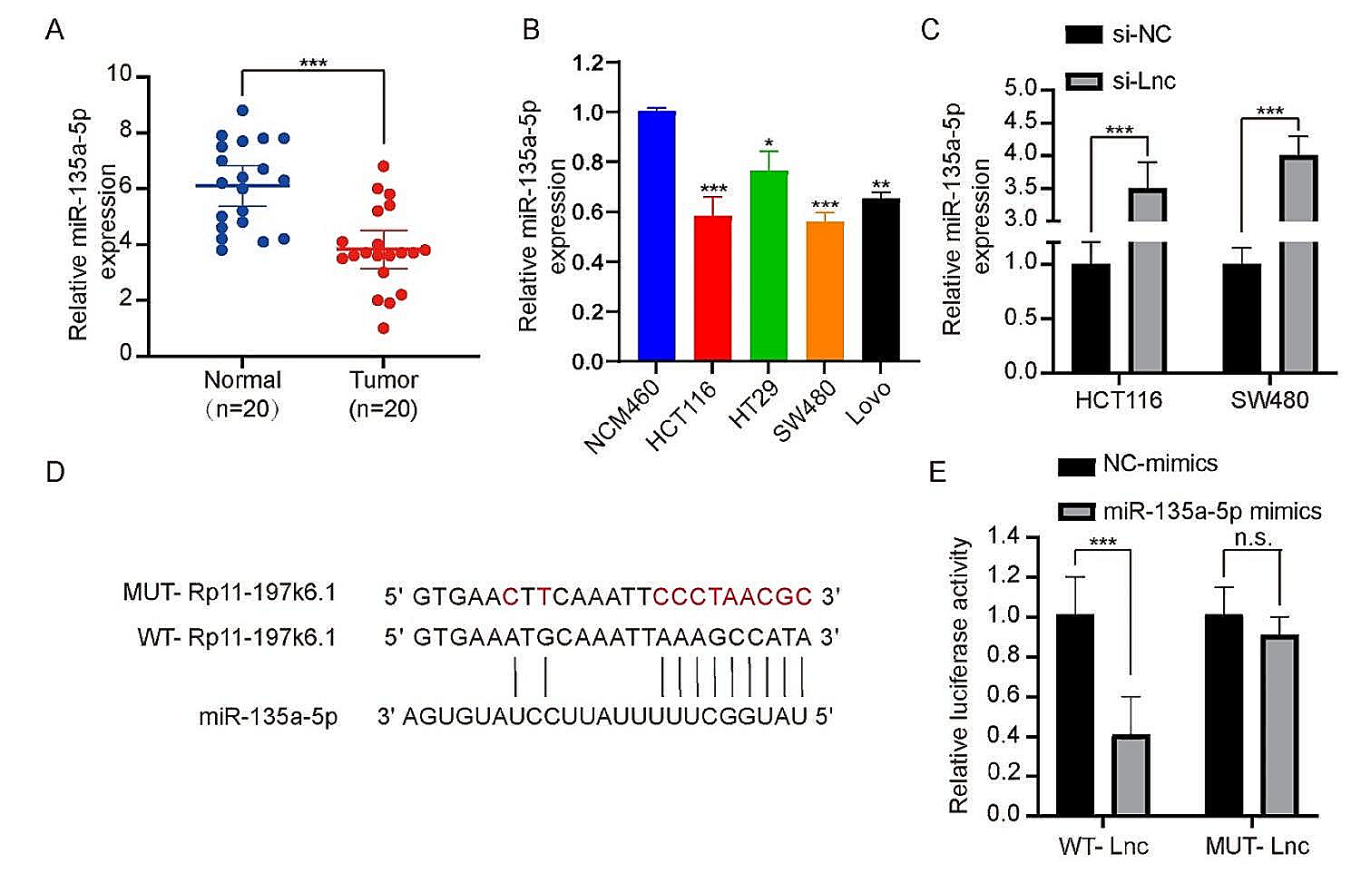
Analysis of miR-135a-5p expression and interaction with lncRNA RP11-197K6.1. **A**: Comparison of miR-135a-5p expression levels between CRC tissues and normal tissues showed elevated miR-135a-5p levels in CRC tissues. **B**: Comparison of miR-135a-5p expression levels across various CRC cell lines (HCT116, HT-29, LOVO, and SW480) and the control cell line NCM460 showed differential expression patterns. **C**: Effect of lncRNA RP11-197K6.1 knockdown on miR-135a-5p expression in HCT116 and SW480 cell lines; changes were observed in miRNA levels post-knockdown. **D**: Bioinformatics analysis revealed the predicted complementary binding sites between lncRNA RP11-197K6.1 and miR-135a-5p, thus supporting the hypothesis of a direct interaction. **E**: Dual-luciferase reporter assay showed that miR-135a-5p reduces luciferase activity in HCT116 cells transfected with the wild-type lncRNA RP11-197K6.1 construct, thus confirming a direct interaction between lncRNA RP11-197K6.1 and miR-135a-5p (n.s.: *P* > 0.05, *P < 0.05, **P < 0.01, ***P < 0.001, *n* = 3)

To investigate the regulatory mechanism of lncRNA RP11-197K6.1 in CRC, given that lncRNA RP11-197K6.1 is primarily localized in the cytoplasm, we hypothesized that lncRNA RP11-197K6.1 might act as a ceRNA in CRC. We screened several miRNAs (results not shown) and found that the expression level of miR-135a-5p was significantly reduced in CRC tissues as compared to that in normal tissues (Fig. 4A). The CRC cell lines (HCT116, HT-29, LOVO, and SW480) (Fig. 4B) showed a significant decrease in miR-135a-5p expression as compared to the control cell line NCM460. It was also observed that lncRNA RP11-197K6.1 knockdown enhanced the expression of miR-135a-5p in HCT116 and SW480 cells (Fig. 4C), thus further confirming a mutual suppression relationship between lncRNA RP11-197K6.1 and miR-135a-5p. Bioinformatics analysis was performed to predict the complementary binding sites between lncRNA RP11-197K6.1 and miR-135a-5p (Fig. 4D), thus suggesting that miR-135a-5p could be the target miRNA that binds to lncRNA RP11-197K6.1. Dual-luciferase reporter assay showed that the overexpression of miR-135a-5p reduced the luciferase activity of HCT116 cells transfected with wild-type lncRNA RP11-197K6.1, but did not reduce the luciferase activity of HCT116 cells transfected with mutant lncRNA RP11-197K6.1 (Fig. 4E). These results indicated that lncRNA RP11-197K6.1 directly “sponged” miR-135a-5p in CRC.

### Overexpression of miR-135a-5p inhibits the proliferation, migration, and invasion of CRC cells

**Fig. 5:**
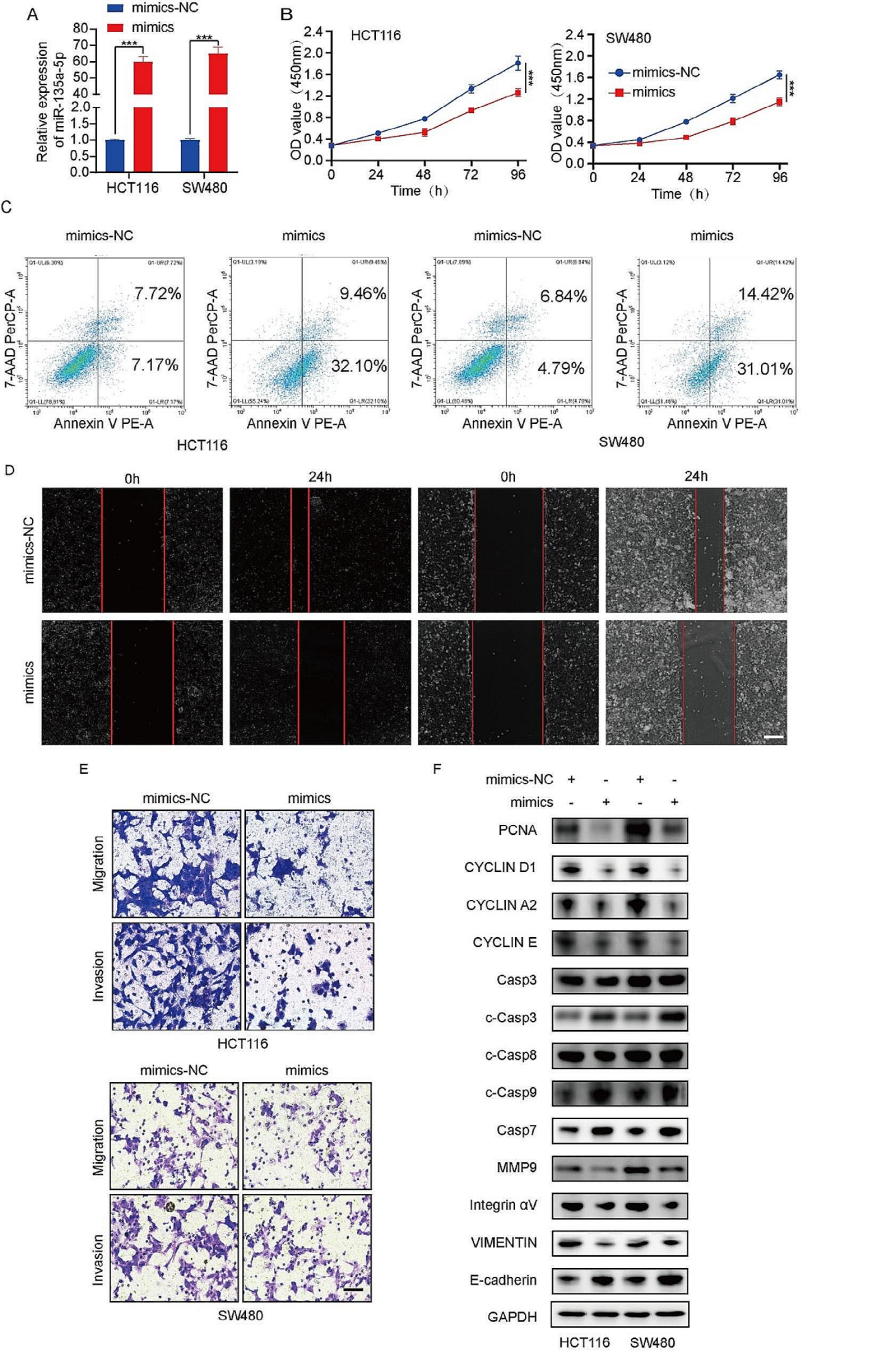
Effects of miR-135a-5p overexpression in CRC cell lines. **A**: Confirmation of transfection efficiency for miR-135a-5p mimics in HCT116 and SW480 cell lines. **B**: Effect of miR-135a-5p overexpression on the proliferation of HCT116 and SW480 cells, indicating reduced proliferation rates. **C**: Increased apoptosis in HCT116 and SW480 cells with miR-135a-5p overexpression, as confirmed by flow cytometry. **D**: Wound healing assays showed decreased migration ability in HCT116 and SW480 cells overexpressing miR-135a-5p (scale = 200 μm). **E**: Transwell assays demonstrated reduced invasion and migration ability of HCT116 and SW480 cells overexpressing miR-135a-5p (scale = 50 μm). **F**: Effects of miR-135a-5p overexpression on the levels of proteins related to the proliferation, apoptosis, and migration of HCT116 and SW480 cells (***P < 0.001, *n* = 3)

Next, we evaluated the role of miR-135a-5p in CRC development. We transfected CRC cells with miR-135a-5p mimics (Fig. 5A). The overexpression of miR-135a-5p using miR-135a-5p mimics significantly inhibited the proliferation, migration, and invasion of HCT116 and SW480 cells and promoted apoptosis of these cells (Fig. 5B-E). The overexpression of miR-135a-5p significantly affected the protein levels of PCNA and MMP9 and increased the protein level of cleaved caspase-3 (Fig. 5F). Thus, miR-135a-5p regulated CRC development, inhibited apoptosis of CRC cells, and promoted the proliferation, migration, and invasion of HCT116 and SW480 cells.

### DLX5 is a direct target of miR-135a-5p

To further investigate the role of miR-135a-5p in CRC and its regulatory network, we focused on DLX5, a protein known to play a key role in the development of various tumors. Previous studies have confirmed that DLX5, a transcription factor, is closely related to tumor progression, and its upregulation in various tumors is usually associated with poor prognosis in patients.

**Fig. 6:**
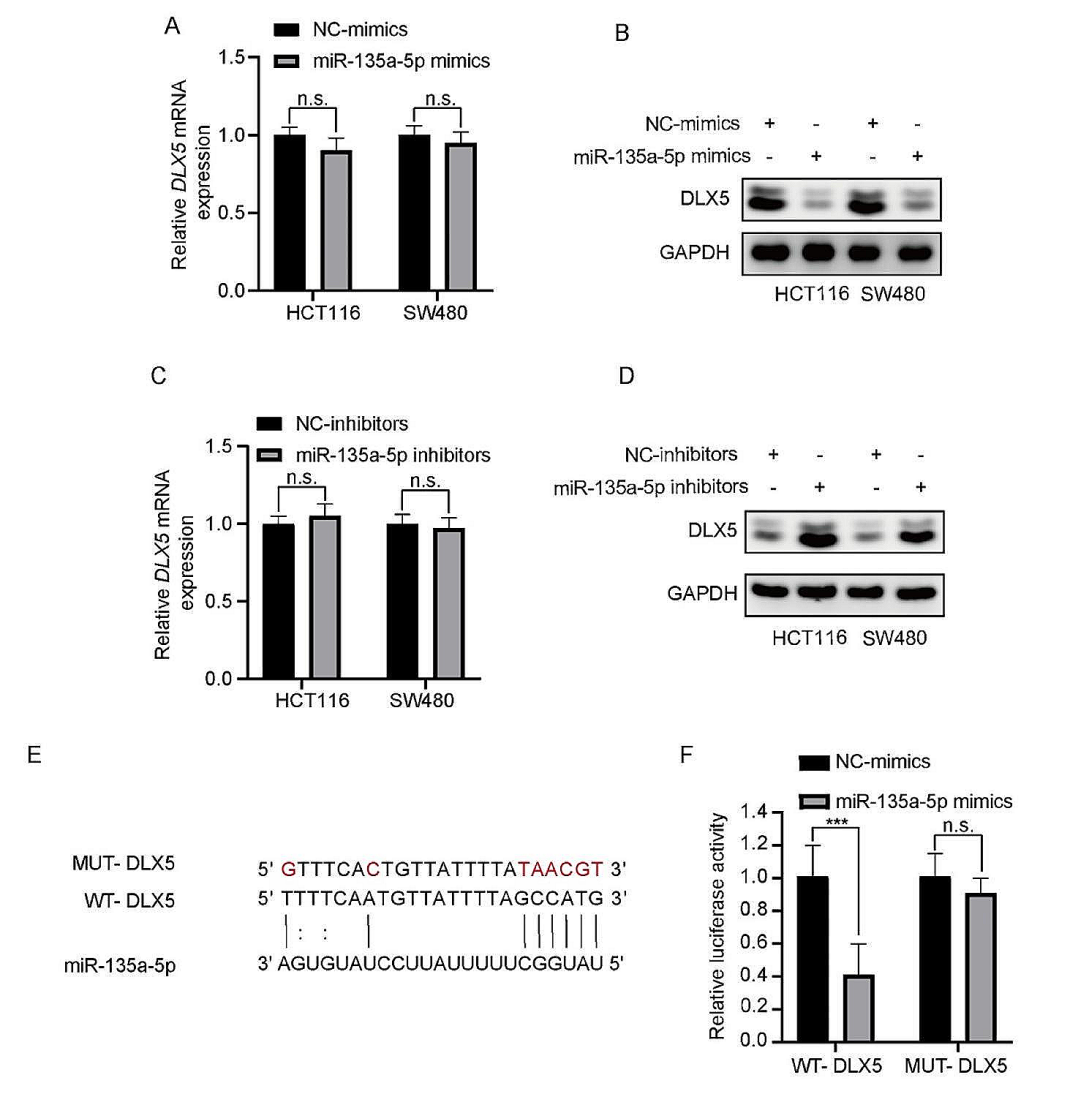
Regulation of DLX5 by miR-135a-5p in CRC cell lines. **A**: RT-qPCR analysis of DLX5 mRNA levels after miR-135a-5p overexpression in HCT116 and SW480 cells. **B**: Western blotting assay of DLX5 protein expression following miR-135a-5p overexpression in HCT116 and SW480 cells. **C**: RT-qPCR analysis of DLX5 mRNA levels after treatment of HCT116 and SW480 cells with the miR-135a-5p inhibitor. **D**: Western blotting assay of DLX5 protein expression following the treatment of HCT116 and SW480 cells with the miR-135a-5p inhibitor. **E**: Bioinformatics analysis using the TargetScan tool predicted DLX5 as a potential direct target of miR-135a-5p based on the binding of miR-135a-5p to the 3ʹ-UTR region of the DLX5 gene. **F**: Dual-luciferase reporter assay confirmed that miR-135a-5p directly targets the 3ʹ-UTR of DLX5, which reduces the luciferase activity of cells transfected with DLX5-WT. No effect was observed in cells transfected with the DLX5 mutant. (n.s.: *P* > 0.05, ***P < 0.001, *n* = 3)

Western blotting assays and RT-qPCR analyses revealed distinct regulatory effects at the transcriptional and translational levels after manipulating miR-135a-5p levels in HCT116 and SW480 cells. In cells transfected with the miR-135a-5p mimic, although DLX5 mRNA levels did not show significant changes (Fig. 6A), the protein expression level was significantly downregulated (Fig. 6B). Conversely, in cells treated with the miR-135a-5p inhibitor, although there was no significant change in DLX5 mRNA levels (Fig. 6C), protein expression was significantly upregulated (Fig. 6D). These results demonstrated that miR-135a-5p predominantly influenced DLX5 expression at the post-transcriptional level, inhibiting its translation in CRC cells. This underscored the direct inhibitory role of miR-135a-5p on DLX5 protein synthesis.

To confirm the direct interaction between miR-135a-5p and DLX5, we conducted a bioinformatics analysis. By using the TargetScan online tool, we found that miR-135a-5p had potential binding sites in the 3ʹ-UTR region of DLX5 (Fig. 6E). To confirm this finding, we performed a dual-luciferase reporter assay by constructing reporter gene vectors containing DLX5 3ʹ-UTR wild-type sequence (DLX5-WT) and mutant sequence (DLX5-Mut). The experimental results showed that miR-135a-5p significantly reduced the luciferase activity of the vector with DLX5-WT, but did not affect the luciferase activity of the vector with DLX5-Mut (Fig. 6F). This result confirmed that miR-135a-5p inhibited the expression of DLX5 by binding to its 3ʹ-UTR region.

### lncRNA RP11-197K6.1 regulates the proliferation, apoptosis, invasion, and migration of CRC cells through miR-135a-5p and DLX5

**Fig. 7:**
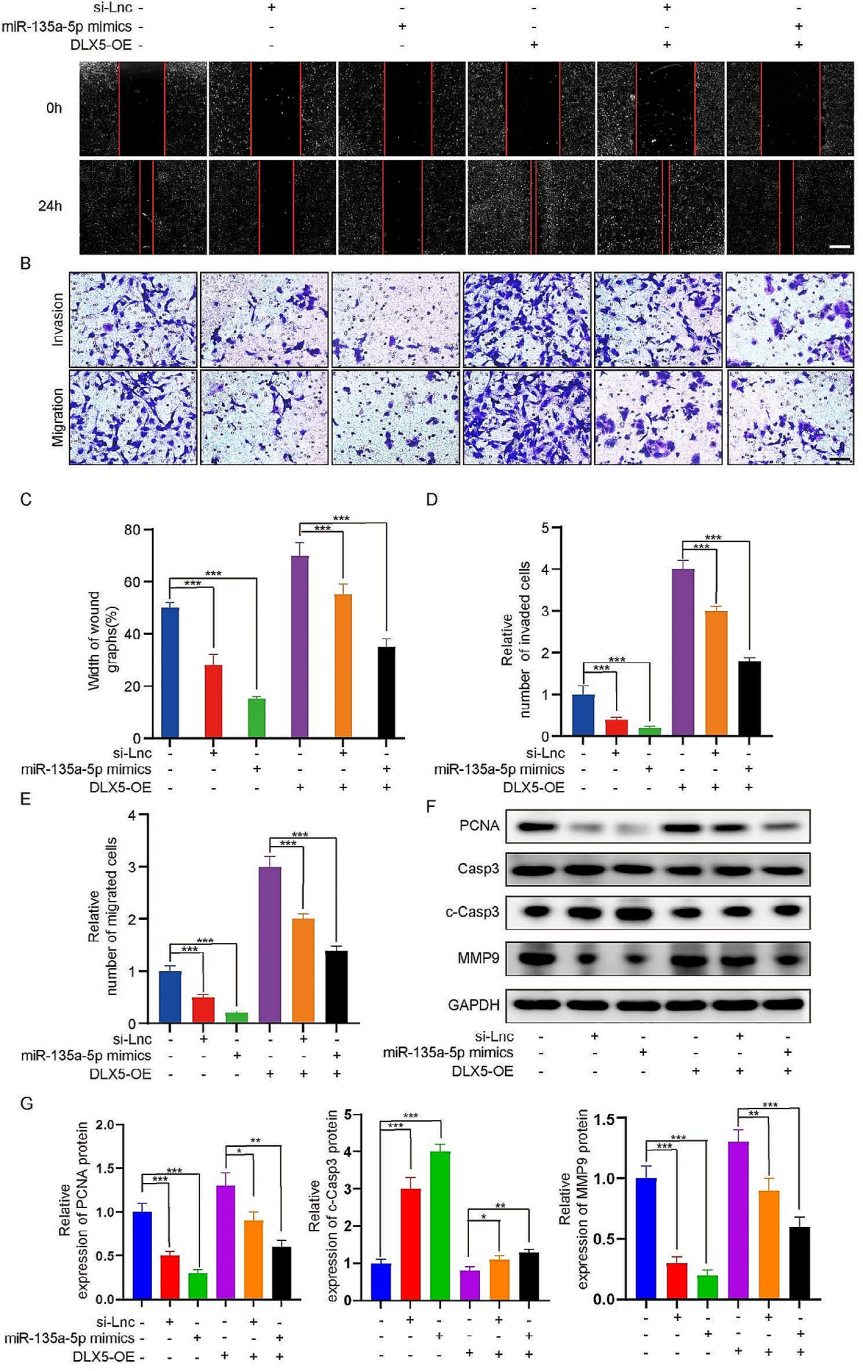
Combined effects of lncRNA RP11-197K6.1 knockdown, miR-135a-5p mimic transfection, and DLX5 overexpression on HCT116 cells. **A**: Wound healing assay to assess the combined effects of lncRNA RP11-197K6.1 knockdown, miR-135a-5p mimic transfection, and DLX5 overexpression on the migration capabilities of HCT116 cells. Sequential images were captured at 0 and 24 h post-wounding (scale = 200 μm). **B**: Transwell assay to evaluate the combined effects of lncRNA RP11-197K6.1 knockdown, miR-135a-5p mimic transfection, and DLX5 overexpression on the invasion and migration of HCT116 cells (scale = 50 μm). **C**: Quantitative analysis of the wound closure rate in the wound healing assay, calculated as the percentage reduction in wound width from initial (0 h) to 24 h post-wounding by using the formula: (Initial Width − 24-h Width) Initial Width × 100%). **D**: Statistical analysis of the invasion data from the Transwell invasion assay, quantified as the number of cells that penetrated the membrane as compared to controls. **E**: Statistical analysis of the migration data from the Transwell migration assay, quantified as the number of cells that migrated through the pores as compared to controls. **F**: Western blotting assay to determine the effects of lncRNA RP11-197K6.1 knockdown on tumor progression markers in HCT116 cells. **G**: Statistical analysis of the data from western blotting assay shown in Fig. 7F (*P < 0.05, **P < 0.01, ***P < 0.001, *n* = 3)

We further investigated the role of lncRNA RP11-197K6.1 in regulating the key processes of tumor development, such as proliferation, apoptosis, invasion, and migration of CRC cells. By using a specific siRNA (si-LncRNA-RP11-197K6.1) to reduce the expression of lncRNA RP11-197K6.1, we observed that the migration and invasion abilities of HCT116 cells were significantly restored when co-transfected with the miR-135a-5p inhibitor or pcDNA DLX5 as compared to that in cells transfected with si-LncRNA-RP11-197K6.1 alone. This result was validated by wound healing assay and Transwell invasion assay; these assays demonstrated the inhibitory effect of lncRNA RP11-197K6.1 knockdown on the behavior of CRC cells and the reversal of this inhibitory effect by the miR-135a-5p inhibitor and pcDNA DLX5 (Fig. 7A-E).

Next, we performed western blotting assay to measure the expression levels of key protein markers associated with tumor progression, including PCNA, MMP9, and cleaved caspase-3 (Fig. 7F). The protein levels of PCNA and MMP9 decreased, while the expression level of the apoptosis marker cleaved caspase-3 increased in cells transfected with si-LncRNA-RP11-197K6.1; this finding indicated that the downregulation of lncRNA RP11-197K6.1 inhibited the proliferation and invasion abilities of CRC cells while promoting their apoptosis. Moreover, following co-transfection with the miR-135a-5p inhibitor or pcDNA DLX5, these changes were partially reversed, leading to increased expression of PCNA and MMP9 and decreased expression of cleaved caspase-3. This result emphasized the mediating role of miR-135a-5p and DLX5 in the regulation of proliferation, apoptosis, invasion, and migration of CRC cells by lncRNA RP11-197K6.1.

### lncRNA RP11-197K6.1 inhibits CRC progression in vivo through miR-135a-5p

**Fig. 8:**
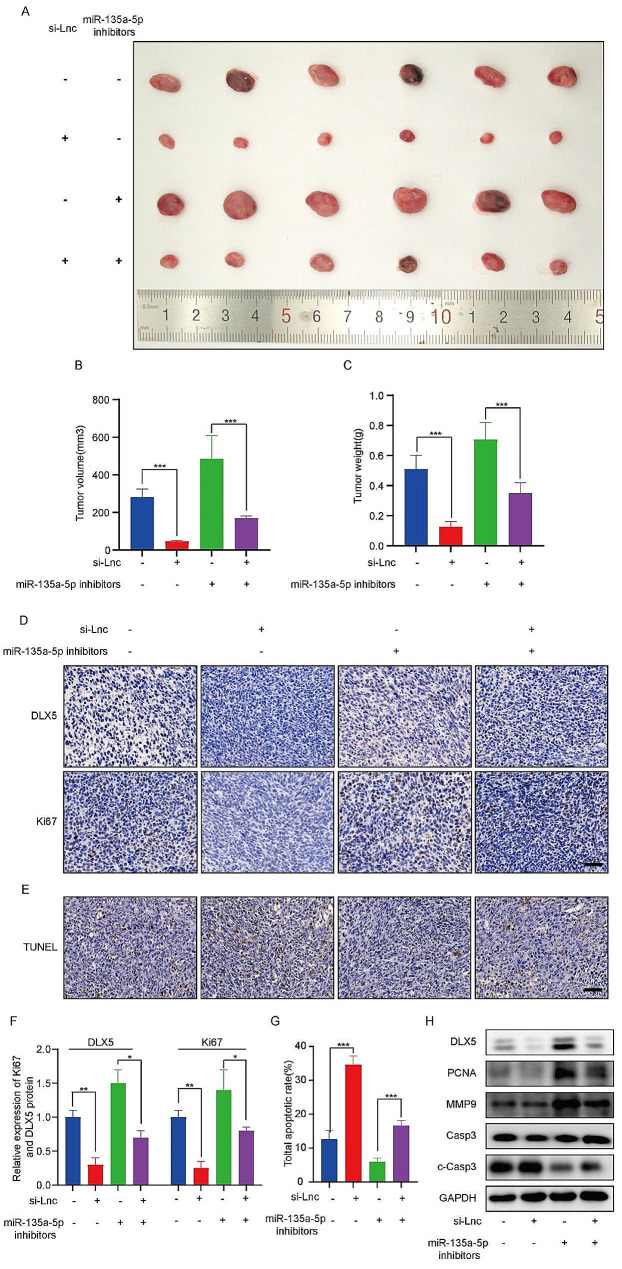
Effects of si-LncRNA-RP11-197K6.1 and the miR-135a-5p inhibitor on a CRC xenograft mouse model with HCT116 cells. **A**: Gross images of tumors from xenograft mice implanted with HCT116 cells transfected with either si-NC (control) or si-LncRNA-RP11-197K6.1. **B**: Bar graph of tumor volumes measured at the endpoint of the study. **C**: Bar graph of tumor weights measured at the endpoint of the study. **D**: Immunohistochemical staining for DLX5 and Ki-67 in tumor tissues from xenograft models. **E**: TUNEL assay results indicated the extent of apoptosis in tumor tissues. **F**: Quantitative analysis of immunohistochemical staining results for DLX5 and Ki-67. **G**: Quantitative analysis of TUNEL assay results. **H**: Western blotting assay showed the effects of si-LncRNA-RP11-197K6.1 and the miR-135a-5p inhibitor on the expression levels of DLX5, PCNA, MMP9, and cleaved caspase-3 in tumor tissues; the results were consistent with in vitro findings (*P < 0.05, **P < 0.01, ***P < 0.001, *n* = 3)

By using a xenograft mouse model, we further studied the function of lncRNA RP11-197K6.1 and miR-135a-5p in CRC in vivo. HCT116 cells transfected with si-NC (negative control) and si-LncRNA-RP11-197K6.1 were implanted into the backs of nude mice to observe tumor growth. The results showed that CRC cells carrying si-NC grew rapidly in mice over time, while the weight and volume of tumors formed by CRC cells carrying si-LncRNA-RP11-197K6.1 were significantly reduced, thus highlighting the critical role of lncRNA RP11-197K6.1 in promoting CRC growth (Fig. 8A-C).

Interestingly, the use of the miR-135a-5p inhibitor reversed the inhibitory effect of si-LncRNA-RP11-197K6.1 on tumor growth, further corroborating the mediating role of miR-135a-5p in the regulation of CRC growth by lncRNA RP11-197K6.1 (Fig. 8A-C).

We performed an immunohistochemical assay to detect the expression levels of DLX5 and the proliferation marker Ki-67 in tumor tissues (Fig. 8D, F); we found that si-LncRNA-RP11-197K6.1 reduced the expression of DLX5 and Ki-67, while the miR-135a-5p inhibitor suppressed the changes induced by si-LncRNA-RP11-197K6.1. These results not only demonstrated the function of lncRNA RP11-197K6.1 in CRC through the miR-135a-5p/DLX5 axis but also highlighted the role of DLX5 as a key factor in tumor growth.

Additionally, we conducted the TUNEL assay to evaluate the extent of apoptosis in the tumor tissues (Fig. 8E, G). The TUNEL assay showed a significant increase in apoptotic cells in tumors treated with si-LncRNA-RP11-197K6.1 as compared to that in controls, thus demonstrating the proapoptotic effect of lncRNA RP11-197K6.1 knockdown. Importantly, the addition of the miR-135a-5p inhibitor reversed the increase in apoptosis induced by si-LncRNA-RP11-197K6.1, thus highlighting its role in mitigating the apoptotic effects through modulation of the lncRNA RP11-197K6.1 pathway.

Finally, we confirmed the existence of this regulatory network by conducting western blotting assay. The results showed that the changes in the expression of DLX5 were consistent with the findings of the immunohistochemical assay; moreover, the changes in the expression of PCNA, MMP9, and cleaved caspase-3 were consistent with in vitro experimental results. These findings highlighted the role of lncRNA RP11-197K6.1 as a key regulator in CRC progression, which functioned as a ceRNA to modulate the activity of miR-135a-5p and its downstream target DLX5. lncRNA RP11-197K6.1 acted as a molecular sponge by effectively sequestering miR-135a-5p and preventing its interaction with DLX5 mRNA. This inhibition of miR-135a-5p allowed for the increased translation of DLX5, which enhanced DLX5 expression. By upregulating DLX5 expression, lncRNA RP11-197K6.1 contributed to the complex regulatory network that drove CRC progression.

## Discussion

There is increasing evidence that dysregulation of lncRNAs affects the tumorigenesis and metastasis of various cancers [19, 20]. lncRNAs play a crucial role in regulating tumor-related genes and influencing the proliferation, migration, and invasion of cancer cells. Current research has clarified that lncRNAs can regulate mRNA expression by competitively binding to miRNAs that target mRNAs [21]. Therefore, lncRNAs are promising biomarkers for cancer diagnosis and can serve as potential therapeutic targets. Lin et al. found that lncRNA FGD5-AS1 accelerated the proliferation of pancreatic cancer cells by regulating the miR-520a-3p/KIAA1522 axis [22], Pan et al. observed that lncRNA DLGAP1-AS1 regulated the development of NSCLC through the miRNA-193a-5p/DTL axis [23]. Thus, lncRNAs are promising diagnostic biomarkers and therapeutic targets.

Genomics and transcriptomics analyses with next-generation sequencing have facilitated a comprehensive analysis of CRC. Many lncRNAs are dysregulated in CRC [24]. In the present study, we screened lncRNAs with altered expression in CRC tissues. To the best of our knowledge, this is the first study to report the high expression of lncRNA RP11-197K6.1 in CRC tissues and cell lines. We confirmed that lncRNA RP11-197K6.1 was an oncogenic lncRNA that promoted CRC progression. Based on RNA sequencing data from the TCGA dataset, we observed that lncRNA RP11-197K6.1 was dysregulated in CRC tissues. We then validated the high expression of lncRNA RP11-197K6.1 in CRC cell lines and tissues through RT-qPCR. We further demonstrated the association of lncRNA RP11-197K6.1 with CRC progression by performing CCK-8 assay, flow cytometry, and Transwell and wound healing assays.

An increasing number of studies have emphasized that the diverse biological functions of lncRNAs largely depend on their unique subcellular localization [25, 26]. Cytoplasmic lncRNAs can regulate mRNA stability or translation and affect the signaling pathways by acting as miRNA decoys [27–29]. By performing FISH experiments and nuclear-cytoplasmic separation experiments, we found that lncRNA RP11-197K6.1 was mainly located in the cytoplasm. Subsequently, we used the TargetScan online tool and discovered that lncRNA RP11-197K6.1 acted as a molecular sponge through the ceRNA mechanism to regulate miR-135a-5p, thereby counteracting the effect of miR-135a-5p on CRC development in *vitro*.

Based on the data from the TCGA database, we found that DLX5 expression was positively correlated with lncRNA RP11-197K6.1. We also noted that DLX5 played an important role in the progression of CRC, was highly expressed in tumor tissues, and was negatively correlated with the overall survival rate. GSEA revealed that the pathways associated with tumor progression, including the hypertrophic cardiomyopathy (HCM) pathway, were significantly enriched. However, our primary focus was on pathways that directly participated in tumor biology. The involvement of the HCM pathway highlighted the complex interactions among various cellular mechanisms, such as cell cycle regulation, cell proliferation, apoptosis, and fibrosis [30–32]. These pathways are crucial in cancer development. Both cardiovascular diseases and cancer lead to the disruption and pathological alteration of vascular structures, which are the key processes in the progression of HCM and tumors [33]. By investigating the relationships between these molecules and pathways, we can gain a deeper understanding of the mechanisms that drive CRC progression and identify vital clues for developing novel therapeutic strategies.

We confirmed the direct binding relationship between lncRNA RP11-197K6.1 and miR-135a-5p through a dual-luciferase reporter system. We then searched for the downstream targets of miR-135a-5p and found that the expression of DLX5 was negatively correlated with miR-135a-5p and positively correlated with lncRNA RP11-197K6.1. DLX5 plays an important role in tumor proliferation and metastasis and is upregulated in various cancers. Previous studies have confirmed that the overexpression of DLX5 can promote tumor cell growth by directly targeting the c-MYC gene [18]. However, the role and expression of DLX5 in CRC have not yet been reported; in the present study, by conducting qPCR and western blotting assay, we found that DLX5 was upregulated in CRC cell lines (HCT116 and SW480) as compared to that in the normal colon epithelial cell line (NCM460).

We hypothesized that lncRNA RP11-197K6.1 regulated DLX5 by sponging miR-135a-5p. The luciferase reporter gene analysis revealed that DLX5 bound to miR-135a-5p. Moreover, the expression of DLX5 was downregulated by miR-135a-5p mimics and upregulated by the miR-135a-5p inhibitor. We proved that inhibiting the expression of miR-135a-5p partially rescued the inhibitory effect of lncRNA RP11-197K6.1 on DLX5. We also further confirmed tumor progression-related proteins through western blotting assay. These results are consistent with previous studies [17], thus showing that the high expression of DLX5 was usually associated with tumor migration and invasion. In summary, our study results revealed a new molecular mechanism for CRC development. The mutual regulation of the lncRNA RP11-197K6.1/miR-135a-5p/DLX5 axis promoted CRC progression. The discovery of this signaling pathway provided new insights into the mechanism of CRC progression and indicated a promising therapeutic target for CRC treatment.

In future studies, given that the role of the lncRNA RP11-197K6.1/miR-135a-5p/DLX5 axis in CRC has been clarified by this study, it is particularly important to further explore its potential role in other types of cancers. This can not only confirm the universality and importance of this regulatory axis but also reveal new cancer treatment targets. Additionally, the development of potential therapeutic strategies targeting this axis is also a critical direction for future research. The use of small molecule compounds, siRNAs, or CRISPR-Cas9 technology to specifically target lncRNA RP11-197K6.1 or miR-135a-5p or to directly regulate the activity of DLX5 through drug delivery systems could be an effective treatment approach. With the development of personalized medicine and precision medicine, therapeutic strategies based on the lncRNA RP11-197K6.1/miR-135a-5p/DLX5 axis are expected to provide more precise and effective treatment options for CRC and other cancers.

This study not only deepened our understanding of the role of lncRNA RP11-197K6.1 in CRC but also provided valuable clues and directions for future cancer research and treatment strategy development. By conducting more mechanism-based studies and clinical application research on this regulatory axis, we expect to bring new hope to cancer patients soon.

## Conclusion

**Fig. 9:**
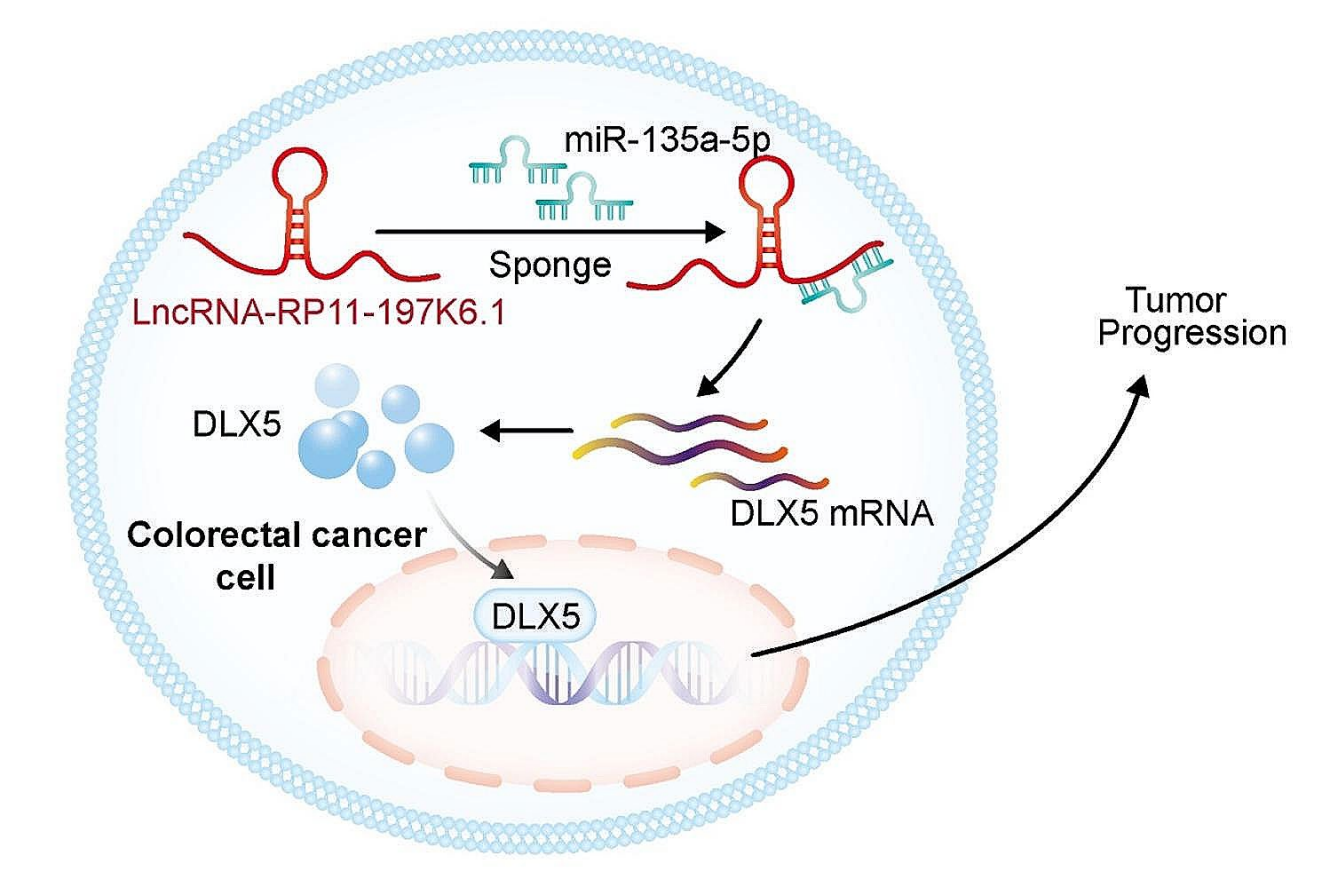
Schematic diagram of mutual regulation among lncRNA RP11-197K6.1, miR-135a-5p, and DLX5

This study revealed the oncogenic role of lncRNA RP11-197K6.1 in CRC progression through a novel regulatory axis involving miR-135a-5p and DLX5. lncRNA RP11-197K6.1 was overexpressed in CRC tissues and cell lines and promoted tumor growth, migration, and invasion while inhibiting tumor cell apoptosis. Mechanistically, lncRNA RP11-197K6.1 acted as a molecular sponge for miR-135a-5p, thereby alleviating miR-135a-5p-mediated suppression of DLX5, a key transcription factor implicated in tumor progression (Fig. 9). The lncRNA RP11-197K6.1/miR-135a-5p/DLX5 regulatory axis provided new insights into the molecular mechanisms that drove CRC progression and highlighted potential therapeutic targets for treating CRC.

### Electronic supplementary material

Below is the link to the electronic supplementary material.

Supplementary Material 1

Supplementary Material 2

Supplementary Material 3

Supplementary Material 4

Supplementary Material 5

## Data Availability

All data generated or analyzed during this study are included in this published article.
