# Supplementary material for: Long non-coding RNA RP11-197K6.1 as ceRNA promotes colorectal cancer progression via miR-135a-5p/DLX5 axis
Source: J Transl Med. 2024 May 17;22:469. doi: 10.1186/s12967-024-05286-5 (PMC11102157; doi:10.1186/s12967-024-05286-5)
Supplement: Supplementary file 1 — Supplementary Material 1 [file 12967_2024_5286_MOESM1_ESM.docx]

| Figure | Comparison | P-value |
| --- | --- | --- |
| Fig 1B | Tumor vs. Normal | <0.001 |
| Fig 1D | HCT116 vs. NCM460 | <0.001 |
|  | HT-29 vs. NCM460 | <0.001 |
|  | Lovo vs. NCM460 | <0.001 |
|  | SW480 vs. NCM460 | <0.001 |
| Fig 2A | si-1 vs. si-nc | <0.001 |
|  | si-2 vs. si-nc | 0.001 |
| Fig 2B | HCT116 si-lnc vs. si-nc | <0.001 |
|  | SW480 si-lnc vs. si-nc | <0.001 |
| Fig 3A | Tumor vs. Normal | <0.001 |
| Fig 3C | Tumor vs. Normal | <0.001 |
| Fig 4A | Tumor vs. Normal | <0.001 |
| Fig 4B | HCT116 vs. NCM460 | <0.001 |
|  | HT-29 vs. NCM460 | 0.031 |
|  | Lovo vs. NCM460 | <0.001 |
|  | SW480 vs. NCM460 | 0.001 |
| Fig 4C | HCT116 si-lnc vs. si-nc | <0.001 |
|  | SW480 si-lnc vs. si-nc | <0.001 |
| Fig 4E | WT-LNC miR-135a-5p vs. NC-mimics | <0.001 |
|  | MUT-LNC miR-135a-5p vs. NC-mimics | 0.3911 |
| Fig 5A | HCT116 mimics vs. mimics-NC | <0.001 |
|  | SW480 mimics vs. mimics-NC | <0.001 |
| Fig 5B | HCT116 mimics vs. mimics-NC | <0.001 |
|  | SW480 mimics vs. mimics-NC | <0.001 |
| Fig 6A | HCT116 miR-135a-5p mimics vs. Nc-mimics | 0.223 |
|  | SW480 miR-135a-5p mimics vs. Nc-mimics | 0.46 |
| Fig 6C | HCT116 miR-135a-5p mimics vs. Nc-mimics | 0.356 |
|  | SW480 miR-135a-5p mimics vs. Nc-mimics | 0.665 |
| Fig 6F | WT-DLX5 miR-135a-5p vs. NC-mimics | <0.001 |
|  | MUT-DLX5 miR-135a-5p vs. NC-mimics | 0.3782 |
| Fig 7C | B vs. A | <0.001 |
|  | C vs. A | <0.001 |
|  | E vs. D | <0.001 |
|  | F vs. D | <0.001 |
| Fig 7D | B vs. A | <0.001 |
|  | C vs. A | <0.001 |
|  | E vs. D | <0.001 |
|  | F vs. D | <0.001 |
| Fig 7E | B vs. A | <0.001 |
|  | C vs. A | <0.001 |
|  | E vs. D | <0.001 |
|  | F vs. D | <0.001 |
| Fig 7G | B vs. A | <0.001 |
| PCNA | C vs. A | <0.001 |
|  | E vs. D | 0.018 |
|  | F vs. D | 0.002 |
| c-Casp3 | B vs. A | <0.001 |
|  | C vs. A | <0.001 |
|  | E vs. D | 0.021 |
|  | F vs. D | 0.002 |
| Mmp9 | B vs. A | <0.001 |
|  | C vs. A | <0.001 |
|  | E vs. D | 0.008 |
|  | F vs. D | <0.001 |
| Fig 8B | B vs. A | <0.001 |
|  | D vs. C | <0.001 |
| Fig 8C | B vs. A | <0.001 |
|  | D vs. C | <0.001 |
| Fig 8F | B vs. A | 0.001 |
| DLX5 | D vs. C | 0.032 |
| KI67 | B vs. A | <0.001 |
|  | D vs. C | 0.026 |
| Fig 8G | B vs. A | <0.001 |
|  | D vs. C | <0.001 |
