# Supplementary material for: Long non-coding RNA RP11-197K6.1 as ceRNA promotes colorectal cancer progression via miR-135a-5p/DLX5 axis
Source: J Transl Med. 2024 May 17;22:469. doi: 10.1186/s12967-024-05286-5 (PMC11102157; doi:10.1186/s12967-024-05286-5)
Supplement: Supplementary file 2 — Supplementary Material 2 [file 12967_2024_5286_MOESM2_ESM.docx]

Table S1:Sequences for miR-135a-5p and miR-135a-5p inhibitor

| Name | Sequences |
| --- | --- |
| miR-135a-5p | UAUGGCUUUUUAUUCCUAUGUGA |
| miR-135a-5p inhibitor | UCACAUAGGAAUAAAAAGCCA |
