# Supplementary material for: Long non-coding RNA RP11-197K6.1 as ceRNA promotes colorectal cancer progression via miR-135a-5p/DLX5 axis
Source: J Transl Med. 2024 May 17;22:469. doi: 10.1186/s12967-024-05286-5 (PMC11102157; doi:10.1186/s12967-024-05286-5)
Supplement: Supplementary file 3 — Supplementary Material 3 [file 12967_2024_5286_MOESM3_ESM.docx]

Table S2: Primer sequences for qPCR

| Genes | Forward (5’-3’) | Reverse (5’-3’) | Tm (℃) |
| --- | --- | --- | --- |
| *U6* | CTCGCTTCGGCAGCACA | AACGCTTCACGAATTTGCGT | 60 |
| *Lnc-RP11-197K6.1* | CCGCTAAGCACTTGAAAGGATTCTG | GGCTGTCTCCCCATTTGTCACT | 66 |
| *GAPDH* | GAAGGTGAAGGTCGGAGTC | GAAGATGGTGATGGGATTTC | 54 |
| *miR-135a-5p* | UAUGGCUUUUUAUUCCUAUGUGA | mRQ 3’ Primer | 65 |
| *DLX5* | TTCCAAGCTCCGTTCCAGAC | CCTGCTCCGTTTCGCTTTCT | 65 |

# *U6,* U6 small nuclear RNA; *GAPDH*, glyceraldehyde-3-phosphate dehydrogenase.
