# Supplementary material for: Long non-coding RNA RP11-197K6.1 as ceRNA promotes colorectal cancer progression via miR-135a-5p/DLX5 axis
Source: J Transl Med. 2024 May 17;22:469. doi: 10.1186/s12967-024-05286-5 (PMC11102157; doi:10.1186/s12967-024-05286-5)
Supplement: Supplementary file 4 — Supplementary Material 4 [file 12967_2024_5286_MOESM4_ESM.docx]

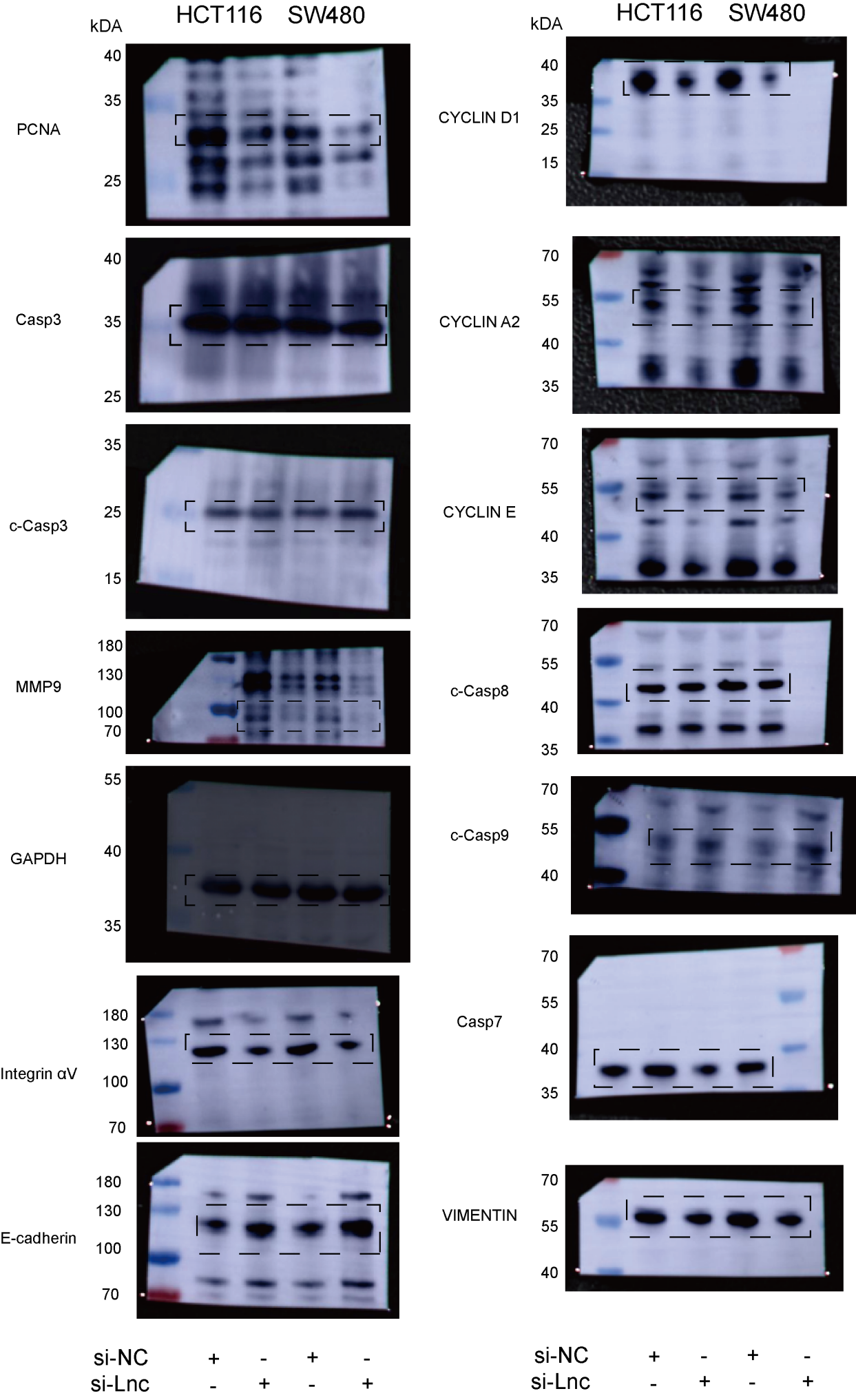


­­Fig. S1. The uncropped images of original western blots in Fig. 2.


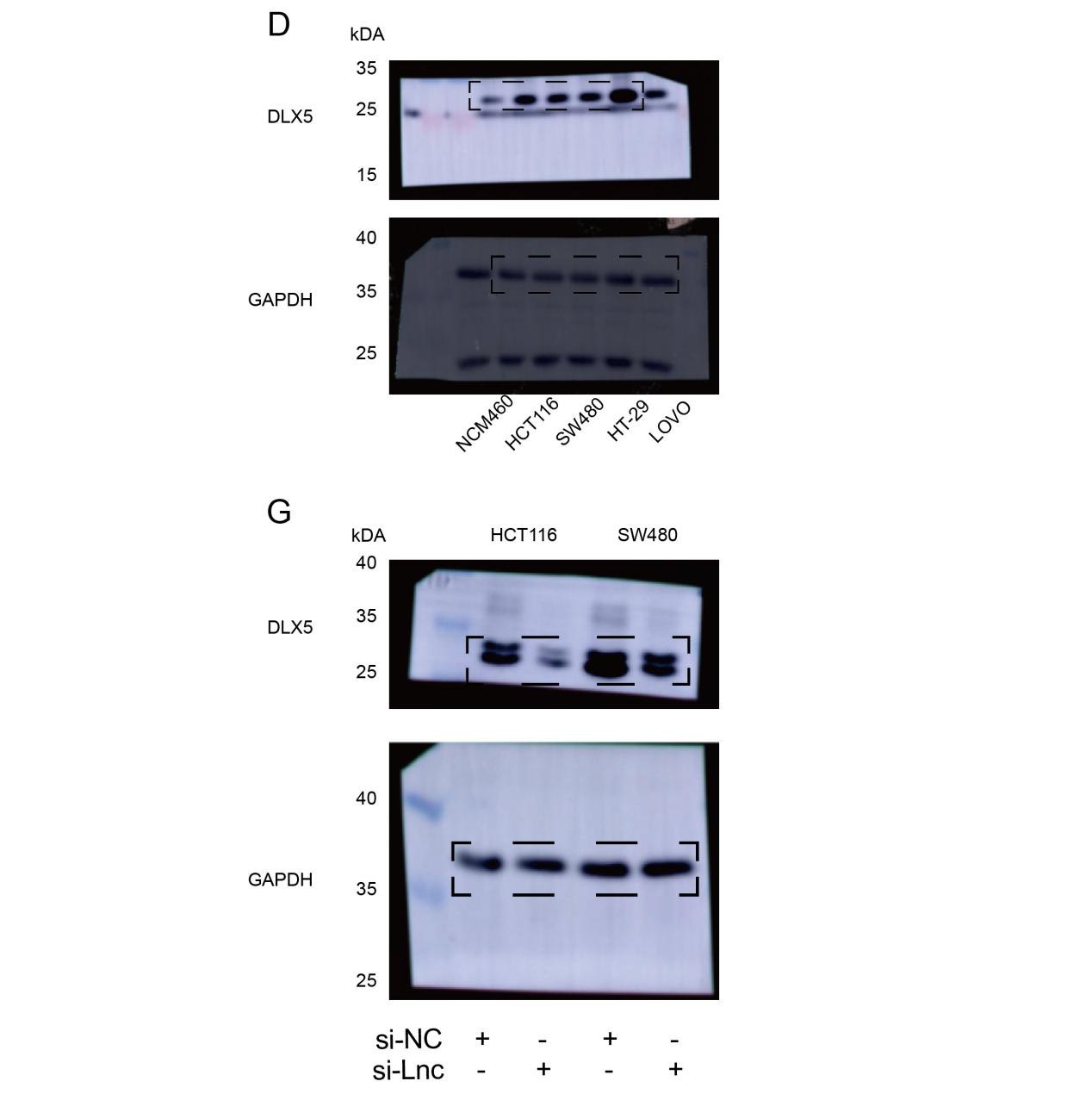


­­Fig. S2. The uncropped images of original western blots in Fig. 3.


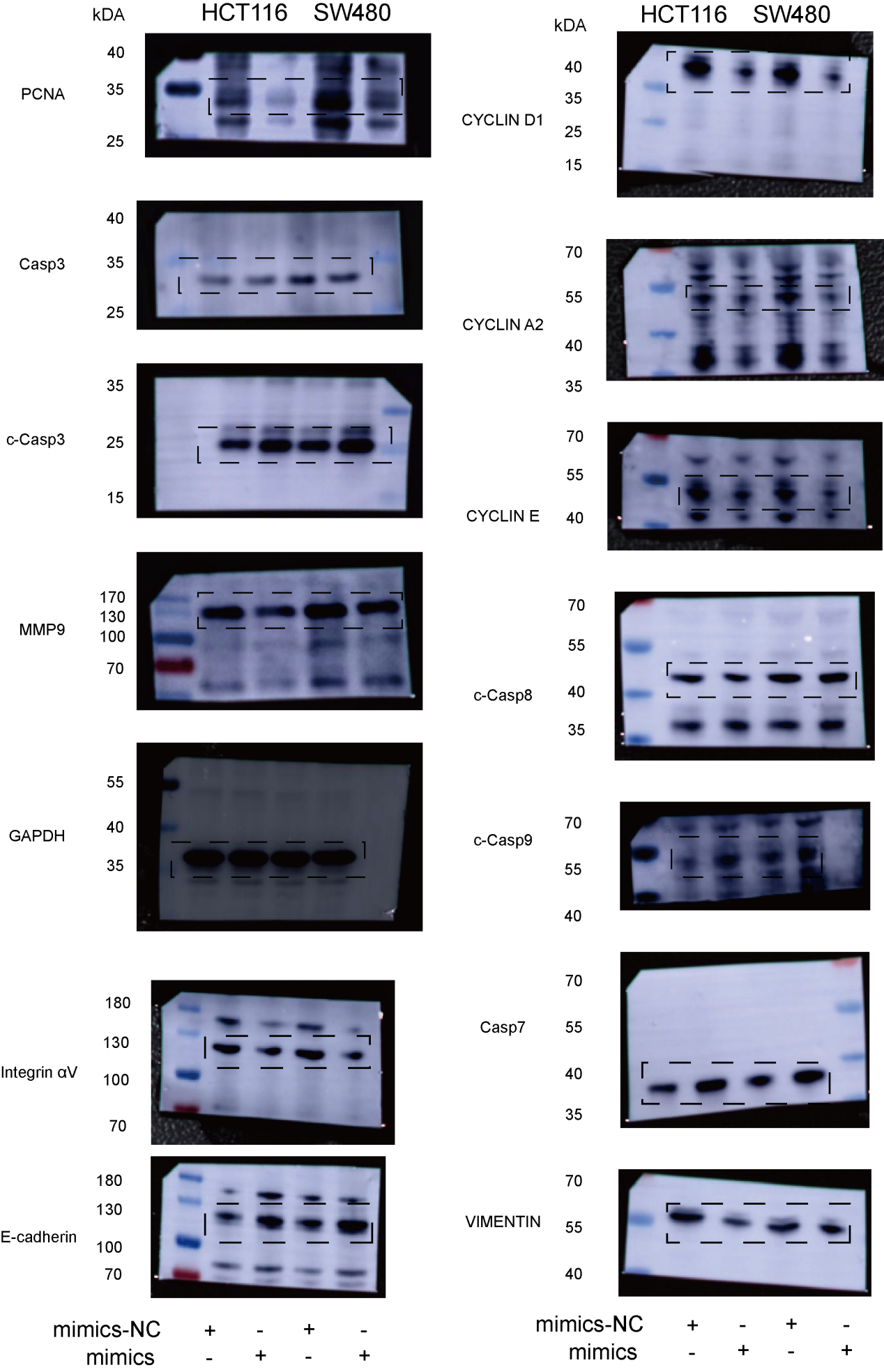


­­Fig. S3. The uncropped images of original western blots in Fig. 5.


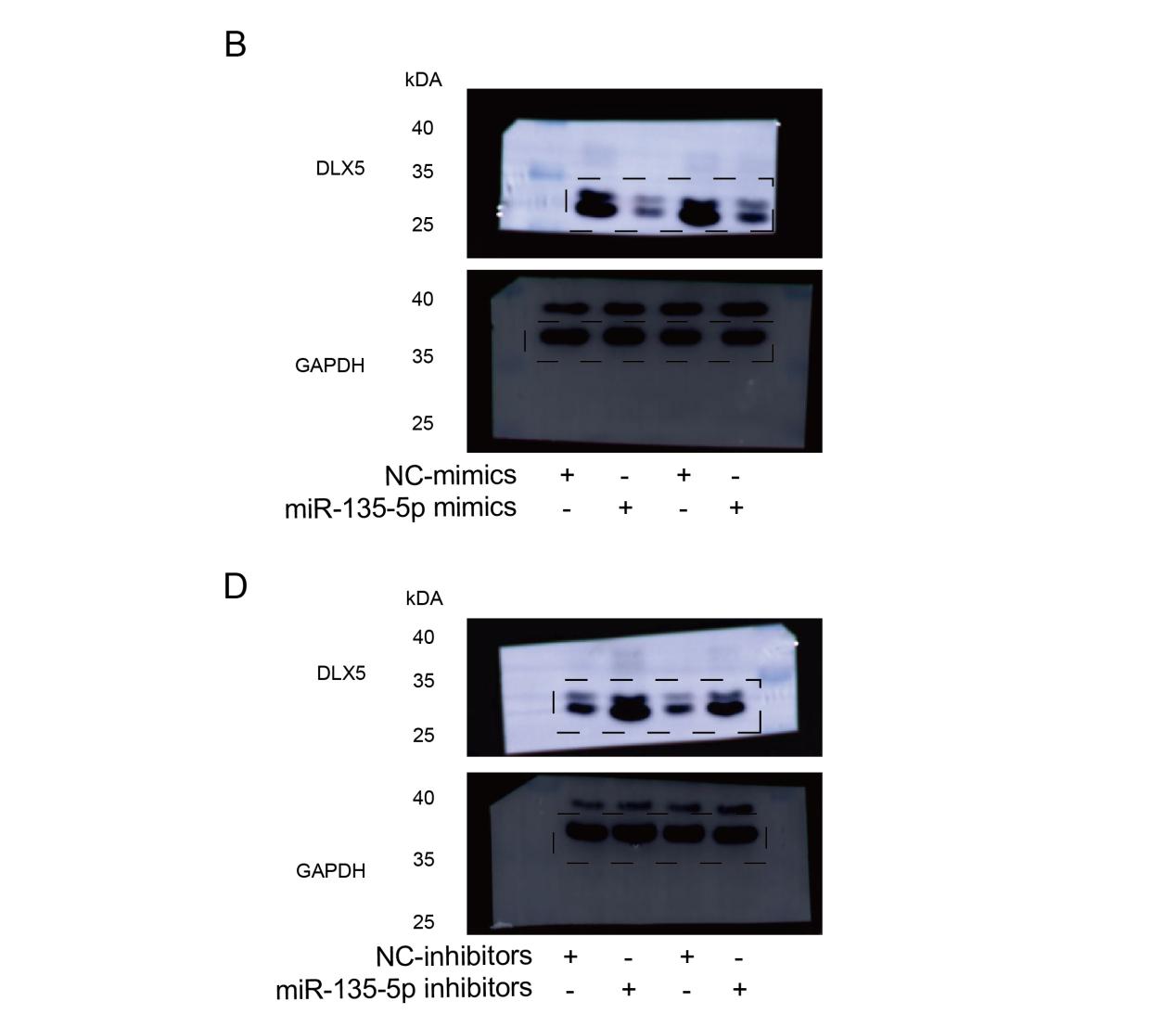
­­

Fig. S4. The uncropped images of original western blots in Fig. 6.


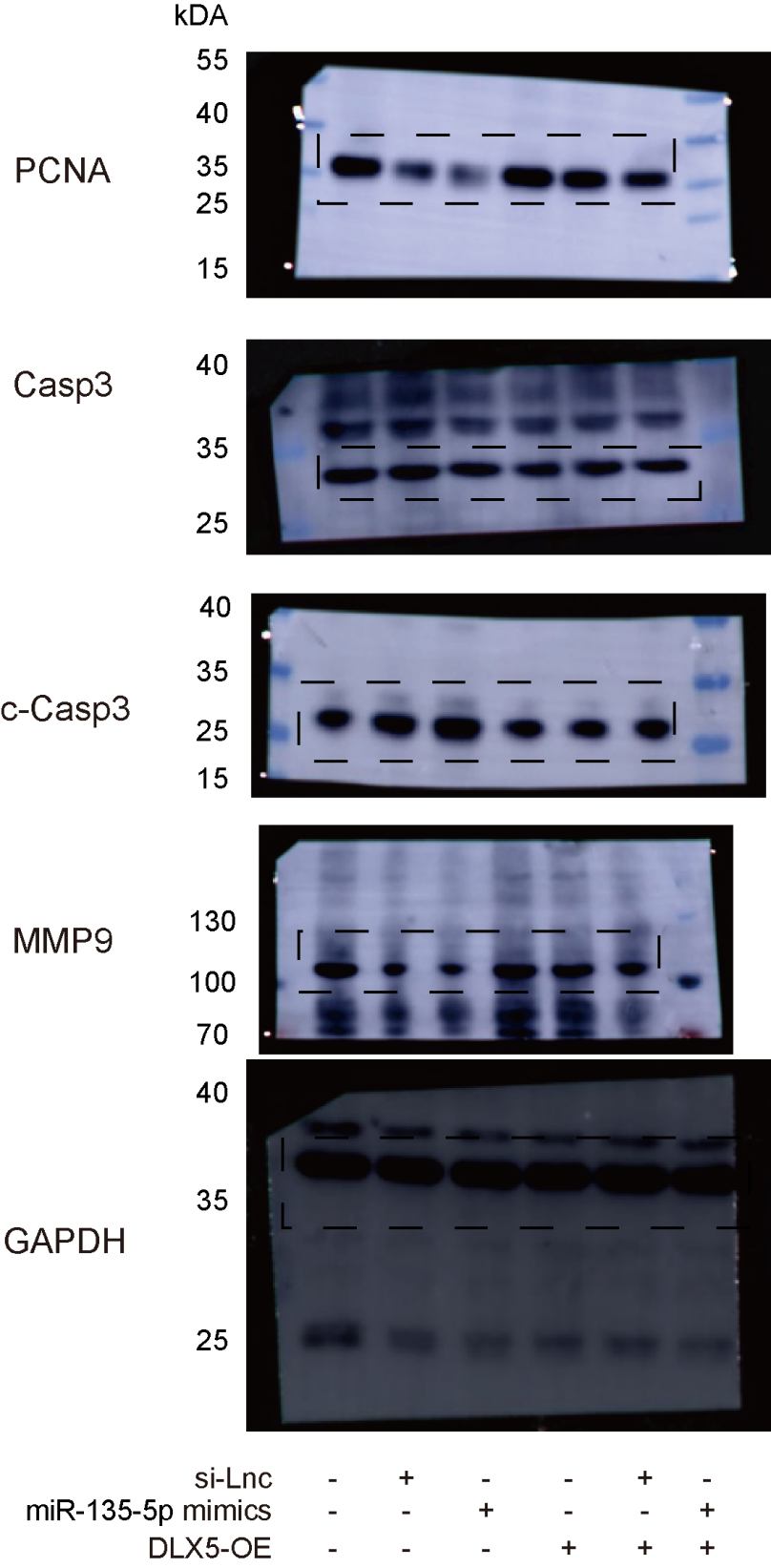


Fig. S5. The uncropped images of original western blots in Fig. 7.


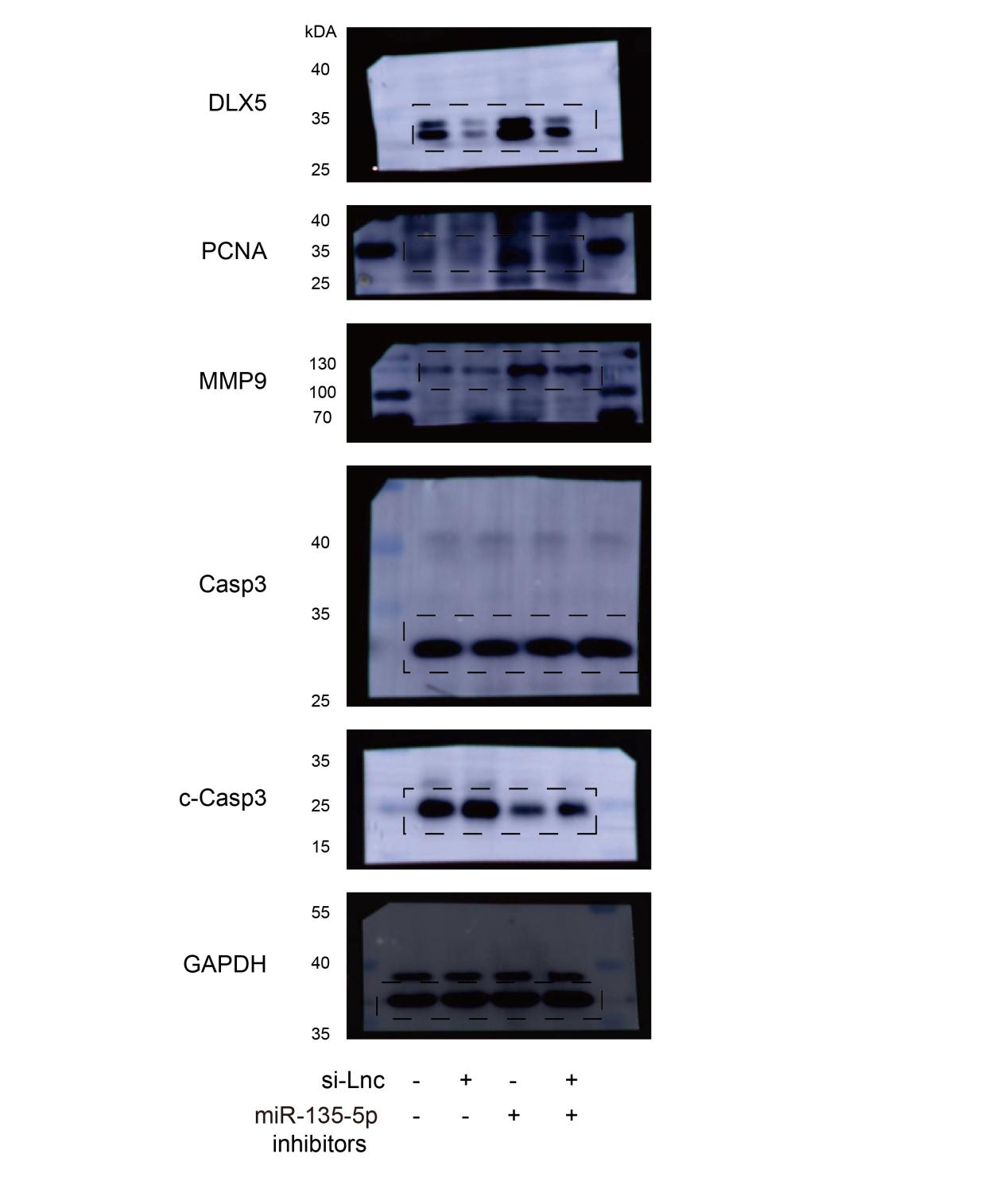


Fig. S6. The uncropped images of original western blots in Fig. 8.
