# Supplementary material for: Long non-coding RNA RP11-197K6.1 as ceRNA promotes colorectal cancer progression via miR-135a-5p/DLX5 axis
Source: J Transl Med. 2024 May 17;22:469. doi: 10.1186/s12967-024-05286-5 (PMC11102157; doi:10.1186/s12967-024-05286-5)
Supplement: Supplementary file 5 — Supplementary Material 5 [file 12967_2024_5286_MOESM5_ESM.docx]

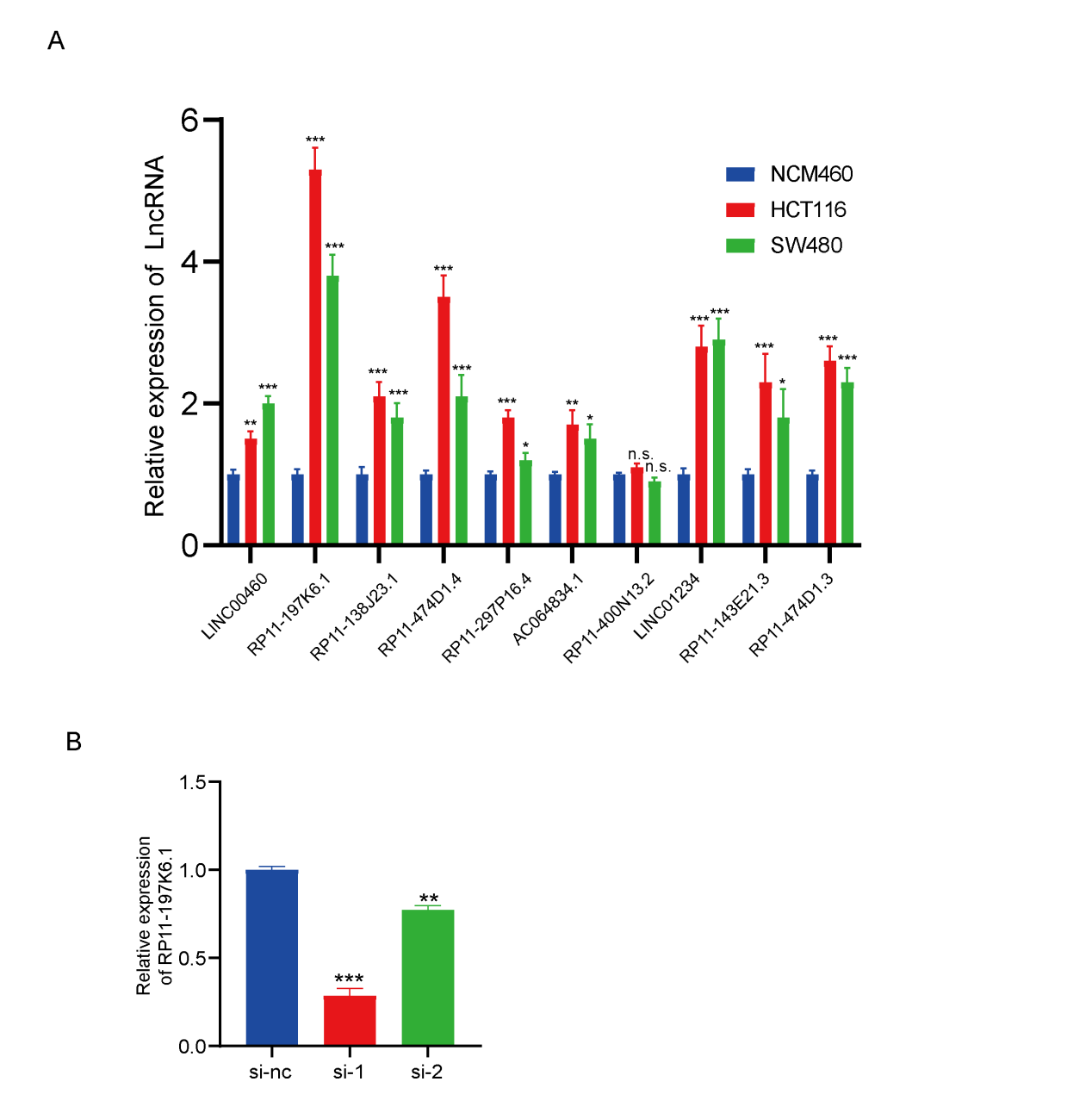


Figure S1: lncRNA expression in CRC cell lines and lncRNA RP11-197K6.1 knockdown efficiency in SW480 cells

A: Comparison of lncRNA expression between CRC cell lines and normal cell lines based on the TCGA-COAD dataset.

A: Confirmation of lncRNA RP11-197K6.1 knockdown efficiency in SW480 cells by RT-qPCR. (n.s.: P > 0.05, *P < 0.05, **P < 0.01, ***P < 0.001, n = 3)
